# On-Surface Synthesis of a Nitrogen-Doped Curved Cycloarene: π‑Extended Pentaazaquintulene and Its Gold Complex

**DOI:** 10.1021/jacs.5c11883

**Published:** 2025-11-11

**Authors:** Zilin Ruan, Olaf A. Kleykamp, Kiyan Linus Haiko Pohl, Tim Naumann, L. Alix Kaczmarek, Anton S. Nizovtsev, Eugen Sharikow, Jörg Sundermeyer, Doreen Mollenhauer, J. Michael Gottfried

**Affiliations:** † 9377 Philipps-Universität Marburg, Fachbereich Chemie, Hans-Meerwein-Str. 4, 35032 Marburg, Germany; ‡ Justus Liebig Universität Gießen, Heinrich-Buff-Ring 17, 35392 Giessen, Germany; § Center for Materials Research (LaMa), 9175 Justus-Liebig University Giessen, 35392 Giessen, Germany; ∥ Helmholtz-Institut für Polymere in Energieanwendungen, Lessingstr. 12-14, 07743 Jena, Germany; ⊥ Institut für Technische Chemie und Umweltchemie, 9378 Friedrich-Schiller-Universität Jena, Philosophenweg 7a, 07743 Jena, Germany; ○ Helmholtz-Zentrum Berlin fur Materialien und Energie GmbH (HZB), 14109 Berlin, Germany

## Abstract

The interplay between π-electron conjugation and molecular geometry has been a central focus in the development of modern hydrocarbon research. Herein, we report the on-surface synthesis and characterization of the nonplanar, π-extended, nitrogen-doped quintulene C85N5 on a Au(111) surface. The as-synthesized C85N5 adopts a convex geometry, which can be inverted to a concave geometry by single-molecule manipulation. In its convex conformation, C85N5 may coordinate a gold atom within its central pore, forming an Au–C85N5 complex through interactions with the pyridinic nitrogen atoms. The structures and electronic properties of these cycloarenes are elucidated by noncontact atomic force microscopy (nc-AFM), scanning tunneling microscopy and spectroscopy (STM/STS), and density functional theory (DFT) calculations. Our results highlight that on-surface synthesis enables the atomically precise generation of curved (hetero)­nanographenes and their metal complexes. Despite the inherent challenges of the nonplanar π-system, both geometric and electronic features can be characterized in detail, and controlled manipulation by scanning probe techniques is feasible.

## Graphical abstract

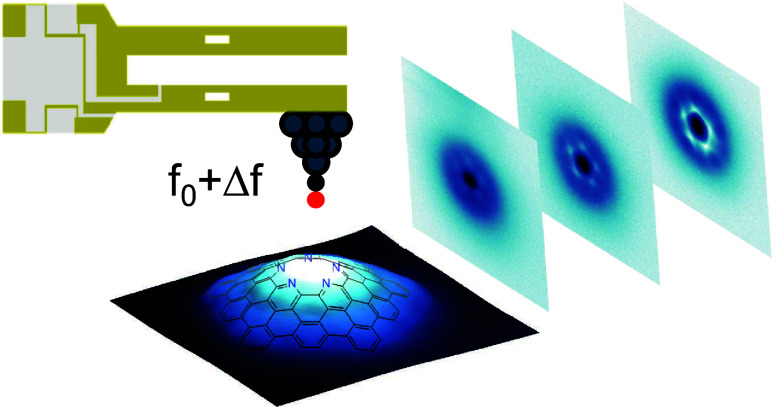

## Introduction

Cycloarenes are a class of annulated macrocyclic π-systems that enclose an inner cavity, typically featuring inward-pointing C–H bonds.^1,2^ Their synthesis was initially motivated by theoretical studies investigating their aromaticity, vibrational frequencies, and magnetic susceptibility.^3–6^ The first and prototypical example of this family is the *D*_*6h*_ symmetric kekulene, synthesized by Staab and Diederich in 1978.^7^ This work is considered as a milestone in cycloarene chemistry, because it provided a general synthetic strategy and raised important questions regarding the electronic structure and π-electron conjugation. Due to synthetic challenges, only a limited number of cycloarenes have been realized since kekulene. These include larger kekulene homologues,^8–10^ expanded kekulene,^11^ heteroatom-doped kekulene,^12,13^ and π-extended kekulenes^14–16^ (Figure 1). The π-extended kekulenes were synthesized by conventional solution-phase chemistry or, more recently, by combined in-solution/on-surface chemistry. They typically adopt planar geometries due to the hexagonal central cavity, and their electronic properties can be modified through variations in size and edge structure.

**Figure 1:**
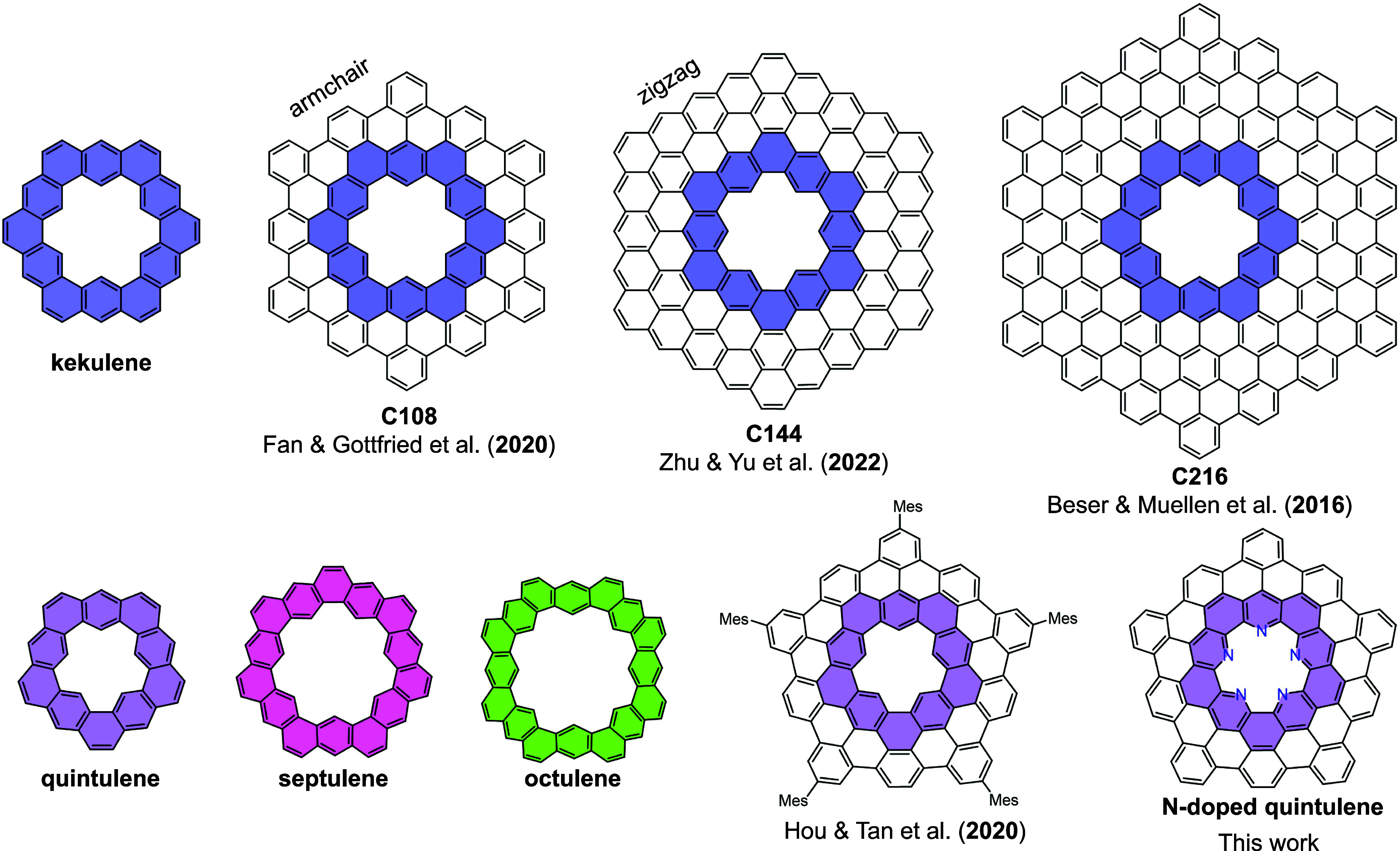
Representative planar cycloarenes, including kekulene, **C108**,^15^ **C144**,^16^ **C216** ^14^ (top row) and curved cycloarenes including quintulene, septulene, octulene, mesityl-substituted extended quintulene,^22^ and the π-extended pentaazaquintulene reported in this work (bottom row).

Inherently nonplanar cycloarenes with pentaradial, heptaradial and octaradial cavity have rarely been achieved due to their highly strained geometry. They belong to the class of nonalternant benzenoid molecules. The nonplanarity of aromatic molecules profoundly influences electronic conjugation, charge transport, and optical properties,^17–19^ making them highly relevant to molecular electronics and materials science. They also serve as molecular models for curved carbon allotropes and as platforms for metal coordination or host–guest interactions,^20,21^ underscoring their significance in developing novel functional materials. Notably, a π-extended mesityl-substituted quintulene^22^ (Figure 1) and a helical quintulene have been synthesized in solution.^23^ However, these molecules tend to dimerize in solution, complicating detailed investigations of their structural and electronic properties.

By contrast, modern on-surface chemistry and scanning probe microscopy techniques have proven highly effective for both the synthesis and characterization of cycloarenes.^24–26^ Planar cycloarenes with various sizes and edge structures, such as **C108** ^15^ and **C144** ^16^ (Figure 1), have been successfully synthesized on surfaces. However, curved cycloarenes have so far remained unrealized in on-surface synthesis, raising critical questions about the feasibility of this approach for highly strained, nonplanar systems. The intrinsic strain in their curved π-conjugated frameworks, driven by pronounced bond torsions and pyramidalization angles, demands precise control over molecular geometry, which is difficult to achieve in surface-mediated reactions. Moreover, the two-dimensional confinement of surfaces restricts molecular flexibility, often favoring planar side products over the desired nonplanar products. These challenges necessitate advanced synthetic strategies, such as optimized precursor designs and transition-metal catalysis, to enable on-surface synthesis of curved cycloarenes. Addressing these obstacles will deepen our understanding of curved π-conjugated systems and broaden the scope of (on-surface) synthetic chemistry for complex molecular architectures.

Here, we demonstrate the synthesis of π-extended pentaazaquintulene **C85N5**, a nonalternant benzenoid, π-extended nitrogen-doped quintulene, by pentamerization of a molecular precursor on a Au(111) surface. The precursor molecule, 4-([1,1’-biphenyl]-2-yl)-2,6-dibromopyridine **1**, undergoes dehalogenative aryl–aryl coupling (Ullmann coupling) to form a cyclo-meta-pentapyridine core **2**, which can coordinate with gold atoms from the surface. Subsequent cyclodehydrogenation leads to the formation of an extended pentaazaquintulene nanocone **3**, which may retain a trapped gold adatom inside its central cavity, depending on its orientation on the surface. Furthermore, the obtained π-extended pentaazaquintulene can be inverted into a bowl-shaped conformation on the surface by STM tip manipulation. The structural and electronic properties of the products were investigated by scanning tunneling microscopy/spectroscopy (STM/STS), noncontact atomic force microscopy (nc-AFM), and density functional theory (DFT) calculations.

## Results and Discussion

The precursor molecule **1** was synthesized in solution starting from 2,6-dibromopyridine **(M1)** in three steps (Figure 2a). **M1** was converted to the boronic acid pinacol ester **M3** via iridium catalyzed borylation.^27,28^ **M3** could not be directly transformed into **1** in a Suzuki-Miyaura cross coupling reaction, because we have not been able to hydrolyze the boronic pinacol ester under typically used basic conditions. Thus, **M3** was first hydrolyzed in an extra step under acidic conditions to the boronic acid **M4** and subsequently coupled under basic conditions with 2-iodobiphenyl resulting in the final precursor **1**. The detailed reactions steps are provided in the supplementary data. Precursor **1** (Figure 2b) was then sublimed onto the clean Au(111) surface held at 480 K applying the high-dilution conditions,^29^ in order to maximize the yield of cyclic products over polymer chains. The hot surface initiates the homolytic cleavage of C–Br bonds, followed by Ullmann coupling, as shown in the reaction scheme in Figure 2b. Figure 2c shows an STM image of molecular islands composed of cyclic pentameric intermediates **2**, held together by Br···H interactions involving residual bromine atoms. Side products such as polymer chains and hexameric macrocycles can also be found on the surface (see overview image in Supplementary Figure S1). The pentameric intermediates **2** show two different contrasts originating from the cavity, as indicated by white and orange arrows in Figure 2c.

**Figure 2:**
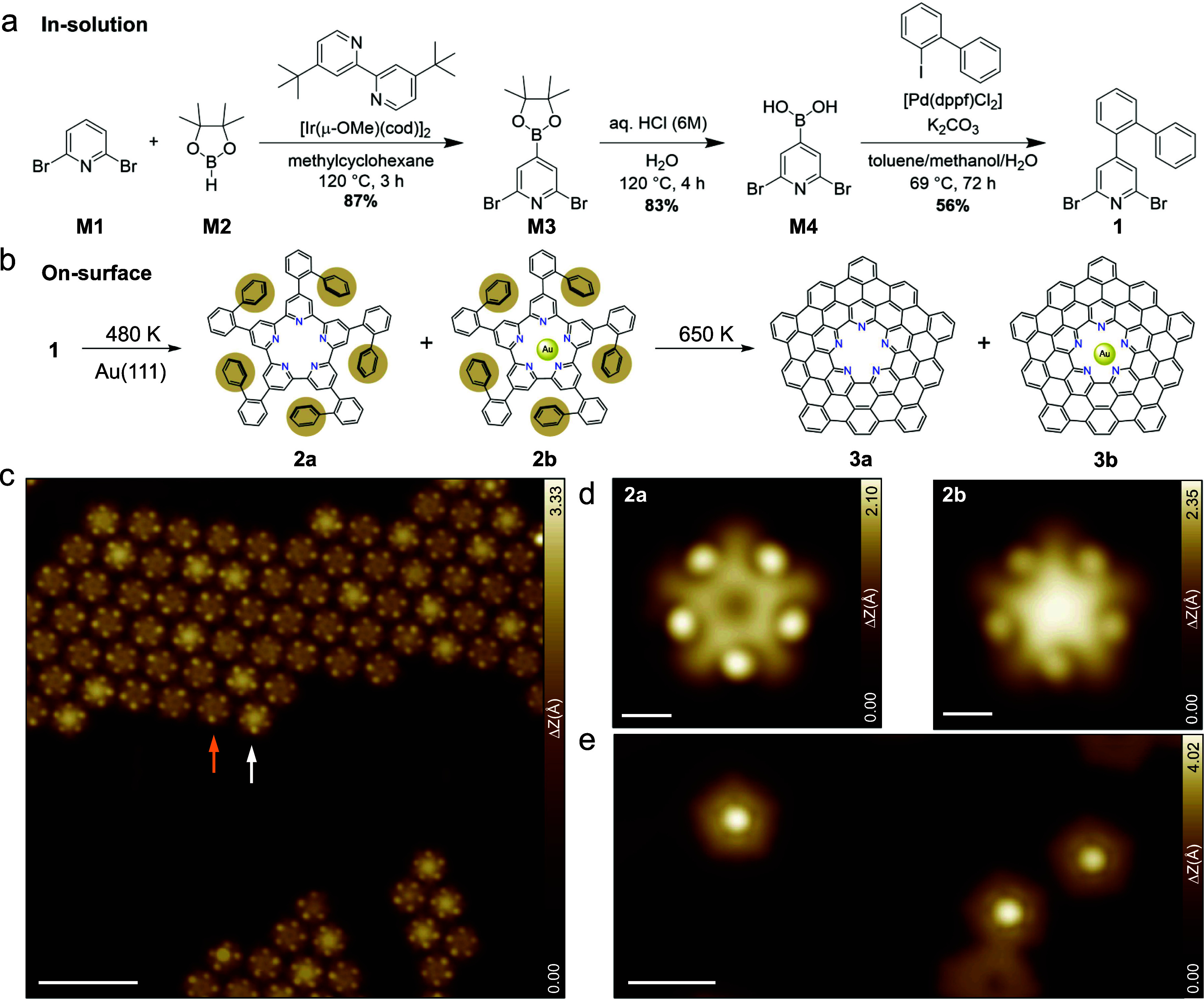
Synthesis of π-extended pentaazaquintulene **3a** and its gold complex **3b**. (a) Solution-phase synthesis of the precursor molecule **1** in a three-step sequence, starting from borylation of 2,6-dibromopyridine (**M1**) followed by acidic hydrolysis and Suzuki-Miyaura cross coupling. (b) On-surface formation of the cyclic pentamer **2a** and its gold complex **2b**, and finally the extended pentaazaquintulene **3a** and its gold complex **3b**. (c) Large-scale STM image showing islands of pentameric intermediates after dehalogenative aryl–aryl coupling (Ullmann coupling) at 480 K. The orange and white arrows denote the pentameric intermediates with empty and filled cavity, respectively. (d) Magnified STM images of the two pentameric intermediates **2a** and **2b**. (e) STM images of the surface after cyclodehydrogenation at 650 K, nonplanar pentagonal molecules (**3a**/**3b**) are observed. Scanning parameters: (c, e) V_s_ = 0.15 V, I_t_ = 30 pA; (d) V_s_ = 0.10 V, I_t_ = 50 pA. Scale bars: (c) 6 nm; (d) 0.5 nm; (e) 2 nm.

A closer inspection of the two pentameric species reveals the presence of five protrusions (Figure 2d) arising from the upward-tilted phenyl groups (Figure 2b, compounds **2a** and **2b**), and a planar center that appears either empty or filled (Figure 2d). We attribute the pentameric intermediate with a filled cavity to complexation with a gold adatom, as expected for the pore with five inward-pointing pyridinic nitrogen atoms. Similar complexation has also been observed for the hexameric intermediate (Supplementary Figure S1). Further annealing to 650 K promotes cyclodehydrogenation (see also Supplementary Figures S2 and S3 for intermediates at lower temperatures) and leads to isolated pentagonal molecules with increased apparent height, consistent with the formation of a π-extended pentaazaquintulene (Figures 2b and 2e). In addition, defective pentamers and linked nanostructures are observed (see Supplementary Figure S4), the latter likely resulting from intermolecular reactions. We also observed a decrease in the coverage, which implies partial desorption during the high-temperature treatment.

The formation of this strained curved cycloarene **3** (**3a** and **3b**) on the relatively inert Au(111) surface is unexpected,^30^ since it usually requires more reactive surfaces such as Pt(111) to catalyze the full cyclodehydrogenation to yield nonplanar carbon nanostructures.^31,32^ To unambiguously confirm the formation of the extended pentaazaquintulene **3**, we acquired high-resolution STM and nc-AFM images with CO-functionalized tip to assess the molecular structure,^33^ as shown in Figure 3a–c. As expected for the convex (dome-shaped) configuration, the positions of the five nitrogen atoms that are close to the tip are clearly visible in the constant height images (Figure 3b,c), while the hexagonal rings are not resolved.

**Figure 3:**
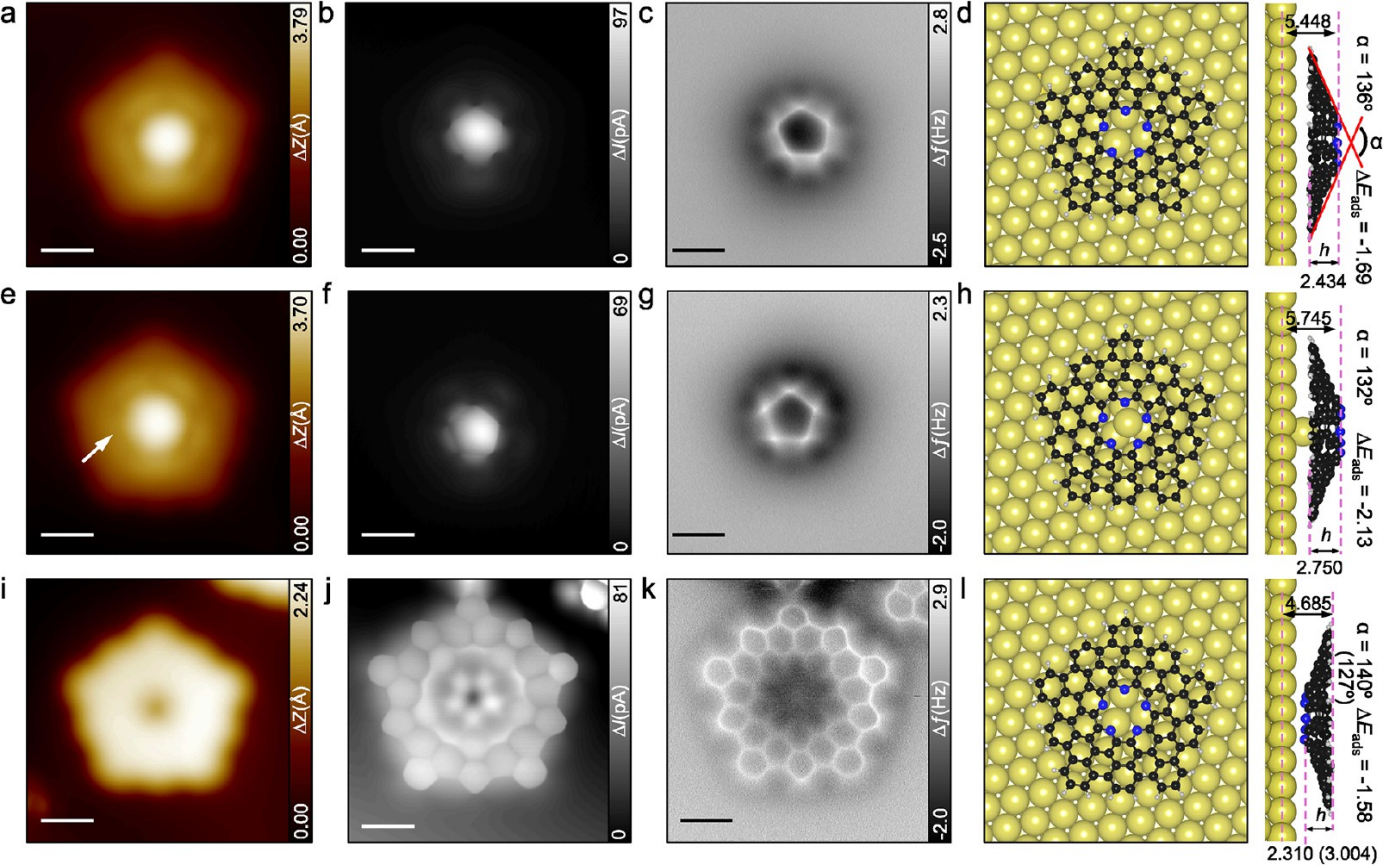
Structural characterization of the π-extended pentaazaquintulene and its Au complex on Au(111). (a–d) Molecule in convex (dome-shaped) geometry. (e–h) Au-complex in convex geometry. (i–l) Molecule in concave (bowl-shaped) geometry. (a, e, i) STM, (b, f, j) BR-STM, (c, g, k) nc-AFM images, and (d, h, l) the DFT optimized models (distances are in Å, adsorption energies are in eV). Scanning parameters: (a, e, i) V_s_ = 0.15 V, I_t_ = 50 pA; (b, c, f, g, j, k) V_s_ = 5 mV. Scale bars: (a-c, e-g, i) 0.6 nm; (j, k) 0.5 nm.

Closer inspection reveals that the corners of the pentaradial pore exhibit identical features, indicating that the extended pentaazaquintulene molecule adsorbs parallel to the surface plane. Coexisting on the surface, some extended pentaazaquintulene molecules show less homogeneous features for the cavity, as highlighted by the white arrow in Figure 3e. The corresponding high-resolution images (Figure 3f,g) reveal similar features for the five inward-pointing nitrogen atoms; however, the overall symmetry is reduced compared to the intrinsic 5-fold symmetry (*C*_5*v*_) observed for the species in Figure 3a–c (see also Supplementary Figure S5), with only a single mirror plane (*C*_*s*_ symmetry) remaining discernible. Furthermore, the frequency-shift versus distance measurements confirms slight variations in the apparent height of the nitrogen atoms (Supplementary Figure S6).

We attribute the two different species to the π-extended pentaazaquintulene with an empty cavity (**3a**) (Figure 3a) and the same molecule with a coordinated gold adatom in the cavity (**3b**, Figure 3e), analogous to the intermediates **2a** and **2b**. The presence of a coordinated gold adatom is further supported by nc-AFM images acquired at closer tip–sample separation both in constant height and constant current mode, in which the metal atom is resolved (Supplementary Figure S5). These observations suggest the formation of coordination bonds between the gold atom and the nearest three nitrogen atoms, which lowers their relative height, leading to the observed 2-fold symmetry. The simulated nc-AFM and bond-resolved STM (BR-STM) images in Supplementary Figures S7 and S8 show excellent agreement with the experimental results, confirming the successful formation of π-extended pentaazaquintulene on the Au(111) surface.

The π-extended pentaazaquintulene synthesized on the Au(111) surface almost exclusively adopts the dome-shaped conformation, implying that this conformation is associated with significantly stronger van der Waals interactions with the surface than the bowl-shaped conformation, contrary to previous reports where bowl-shaped conformations were more favorable on a metallic surface.^34^ Thus, compound **3a**, when confined to the surface, potentially gains an additional structural degree of freedom, exhibiting bistability between dome- and bowl-shaped conformers.

The inversion of the initially formed dome-shaped π-extended pentaazaquintulene **3a** to its bowl-shaped counterpart can be induced by manipulation with the STM tip (Figure 3i, see also Supplementary Figure S9), in a manner similar to that previously demonstrated for sumanene.^34^ Following inversion, the bowl-shaped molecules exhibit significantly higher mobility, further supporting the notion of a weaker surface interaction of the bowl-shaped conformation. Additionally, the bowl-shaped form occasionally appears after annealing at elevated temperatures (Supplementary Figure S10), suggesting that interconversion between the two conformations requires thermal activation.

High-resolution images of the bowl-shaped structure obtained by inversion with the STM tip (Figures 3j and 3k) reveal clearly resolved six-membered rings at the outer edge, while the recessed central region appears darker, indicative of a bowl geometry. Experimental frequency versus distances curves and simulated nc-AFM images (Supplementary Figures S11 and S12) confirm the nonuniform height profile.

To gain mechanistic insight into the inversion process, we located the transition state connecting the dome and bowl conformers at the B3LYP/def2-TZVP level of theory in the gas phase. The transition state was found to be curved, with three nitrogen atoms and adjacent carbon atoms displaced downward, and lies 2.01 eV above the ground state (Supplementary Figure S13). In contrast, a fully planar structure represents a third-order saddle point 3.11 eV above the ground state. Thus, distortion from planarity of the transition state significantly reduces the inversion barrier by 1.10 eV. An even lower barrier is expected for adsorbed π-extended pentaazaquintulene due to interactions with the Au(111) surface.

To gain molecular-level insight into the adsorption process of the observed species, we conducted theoretical calculations focusing on structure and energetics of the adsorbed π-extended pentaazaquintulene. The DFT computations confirm the experimental result that π-extended pentaazaquintulene adopts a nonplanar geometry, giving rise to bowl- and dome-shaped conformations. Although both conformers are equivalent in the gas phase, their minimum energy structures are different upon physical adsorption on the Au(111) surface (Figure 3), as predicted by a computational approach combining semiempirical and dispersion-corrected DFT methods (see computational details in the Supporting Information).

The bowl-shaped conformer resides significantly closer to the metal surface (*d*_Au–N_ = 2.375 Å), interacting with gold atoms primarily via its inner nitrogen-containing ring. This interaction induces a small vertical displacement (indentation) of approximately 0.14 Å of the underlying gold atoms with respect to the topmost gold layer (Figure 3). In contrast, the dome-shaped conformer contacts the surface via its outer rim atoms (*d*_Au–C_ = 3.014 Å), resulting in a larger average molecule–surface separation by 0.639 Å, compared to the bowl. Nevertheless, the dome-shaped conformer has a higher adsorption energy by 0.11 eV, attributable to a higher number of van der Waals contacts with the substrate (Supplementary Table S1).

Furthermore, the calculations show that inclusion of a gold adatom within the cavity of the dome-shaped conformer leads to the formation of a highly stable surface complex (Figure 3h). In this complex, the molecule is laterally displaced relative to the encapsulated Au atom, enabling the formation of three shorter Au–N coordination bonds (Supplementary Table S2) and resulting in reduced symmetry, consistent with the experimental findings. Notably, this dome-shaped conformer binds to Au(111) surface with the Au adatom much stronger than to the pristine Au(111) one (ΔΔ*E*_ads_ = 0.44 eV). According to the performed computational analysis (Supplementary Table S1), which allows to decompose PBE-D4 adsorption energy (Δ*E*_ads_) into PBE and D4 components associated with dispersion interactions (*E*_disp_) and a sum of electrostatic, exchange, and orbital interactions treated by PBE functional (*E*_DFT_), the presence of the Au adatom weakens the dispersion interactions by approximately 0.2 eV, but strengthens the interactions associated with *E*_DFT_ by approximately 0.6 eV.

Structural analysis indicates that both dome-shaped species (with and without the gold adatom) adsorb parallel to the surface, whereas the bowl-shaped conformer is slightly tilted. This tilt arises from differences in adsorption heights among the nitrogen atoms, resulting in unequal N–N distances (Supplementary Table S3). Notably, adsorption induces significant distortion in the π-extended pentaazaquintulene and its gold complex: the benzene rings closest to the surface become flattened, resulting in a more negatively curved structure compared to the naturally conical shape of its mesityl-substituted homologue^22^ and a similar positively curved nanocone based on a corannulene core.^35^ The degree of this distortion is captured by changes in the bending angle α and the molecular depth *h* (Figure 3 and see also Supplementary Figure S14). Adsorption causes α to increase and *h* to decrease, indicating molecular flattening due to surface interaction (Figure 3). The largest deviations from the gas-phase geometry are observed for the bowl-shaped conformer (Δα = 13°, Δ*h* = 0.694 Å), which, interestingly, correlates with its weakest adsorption energy (Δ*E*_ads_ = −1.58 eV). These values are nearly twice those reported previously for a buckybowl molecule adsorbed on Au(111),^36^ highlighting the pronounced adsorption-induced deformation in the present system.

To characterize the electronic properties of the π-extended pentaazaquintulene, we performed scanning tunneling spectroscopy (STS) measurements. Figure 4a shows single point dI/dV spectra acquired at the positions indicated in the inset, revealing narrow peaks in the range of occupied electronic states and broader peaks in the unoccupied range. Notably, the sharp peak around a sample bias of 1 V is attributed to electronic states in the cavity arising from interaction with the surface, rather than intrinsic molecular orbitals (see Supplementary Figures S15–S21). Therefore, we assign the peaks at around −1.62 V and 1.33 V to the highest occupied (HOMO) and the lowest unoccupied molecular orbitals (LUMO), respectively, resulting in a HOMO–LUMO gap of around 2.95 eV. In addition, two peaks at around −2.05 V and 1.64 V, assigned to HOMO–1 and LUMO+1, respectively, are also identified. The corresponding dI/dV maps (Figure 4b), recorded at the energies marked by arrows in Figure 4a, show delocalized electronic states at the inner and outer edges of the molecule. These states are more pronounced for the occupied states.

**Figure 4:**
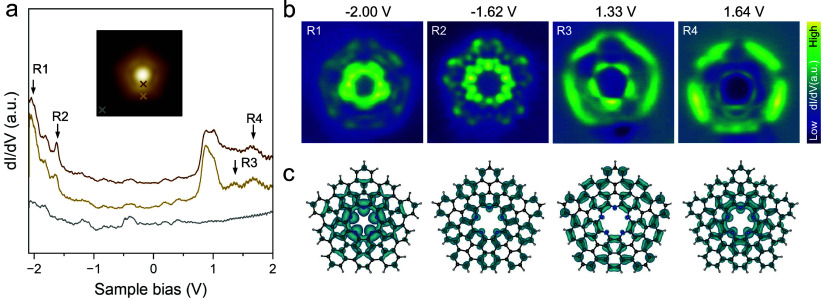
Electronic properties of the π-extended pentaazaquintulene. (a) Point dI/dV spectra acquired at the positions marked by the colored crosses in the inset. (b) Experimental dI/dV maps taken at the energies denoted in (a). (c) The corresponding DFT-calculated orbital density maps, representing the superposition of the squared wave functions of the degenerate orbitals, isovalue = 0.002 *e*/Å^3^. V_rms_ = 20 mV.

For the complex with the coordinated gold adatom, STS spectra reveal a small shift of the energy levels of ∼20 meV toward lower energies, presumably due to interactions between the gold atom and the pyridinic nitrogen atoms (see Supplementary Figure S22). Moreover, the corresponding dI/dV maps (Supplementary Figure S22) exhibit the same 2-fold symmetry as observed in the high-resolution images in Figure 3b,c, confirming the presence of the coordinated gold atom and its influence on the electronic structure of the N-doped extended quintulene. DFT calculations employing both gas-phase and adsorbed (dome- and bowl-shaped) geometries predict a HOMO–LUMO gap of 3.2–3.3 eV at the PBE0 level of theory, suggesting a large quasiparticle gap. These results are consistent with the observed large STS transport gap, taking into account screening effects from the metallic substrate. In addition, they indicate a slight increase in the HOMO–LUMO gap with increasing molecular strain, accompanied by subtle changes in the shape of the molecular orbitals (Supplementary Figures S23–S25).

## Conclusion

In summary, we have demonstrated a combined solution-phase and on-surface synthesis of the π-extended N-doped quintulene C85N5 with a pentaradial cavity. On the Au(111) surface, C85N5 adopts a convex (dome-shaped) geometry, which can be inverted to the corresponding concave (bowl-shaped) geometry by STM-tip manipulation. In its convex conformation, C85N5 forms a coordination complex with a gold adatom, with the metal center ligated by the pyridinic nitrogen atoms decorating the central cavity. The on-surface synthetic route proceeds via a cyclic pentameric Ullmann intermediate, which can likewise host a gold adatom in its central cavity. The curved geometry and the electronic structure of both the quintulene C85N5 and its Au complex have been investigated by nc-AFM, STM, and STS mapping, revealing a HOMO–LUMO gap of 2.95 eV for the extended N-doped quintulene, which is slightly reduced by 0.03 eV upon coordination of a gold adatom. This synthetic approach provides a key advancement toward the bottom-up fabrication of elusive curved cycloarenes and offers fundamental insights into their structural and electronic properties at the single-molecule level.

## Experimental and Computational Details/Methods

### Scanning Probe Microscopy Characterization

Experiments were performed by using an ultrahigh vacuum low temperature-scanning tunneling microscope (UHV LT-STM, Scienta Omicron) with a base pressure better than 1 × 10^–10^ mbar. All the STM images were acquired with constant current or constant height mode by using an electrochemically etched tungsten tip at 4.2 K. All given voltages were applied to the sample with respect to the tip. Nanotec Electronica WSxM software was used to process the images.^37^ Constant current mode nc-AFM measurements were performed at 4.2 K with tungsten tips placed on a qPlus tuning fork sensor^38^ driven at its resonance frequency (26500 Hz) with a constant amplitude of ∼ 70 pm. The tips were functionalized with a single CO molecule at the tip apex^33^ picked up from the Au surface after dosing CO. The Δz was positive (negative) when the tip–surface distance was increased (decreased) with respect to the STM set point at which the feedback loop was opened. The dI/dV spectra were recorded using a lock-in amplifier with a modulation frequency of 579 Hz and an amplitude of 10–20 mV. dI/dV maps were collected in constant-current mode.

### Computational Investigations

#### Periodic DFT Calculations

DFT calculations with periodic boundary conditions were carried out using the Vienna *Ab initio* Simulation Package (VASP 6.3.2).^39–60^ The PBE density functional^41^ with Grimme’s dispersion correction D3­(BJ) was used in combination with the standard PBE projected augmented wave (PAW) potentials^42,43^ with the kinetic energy cutoff set to 425 eV. The equilibrium structures were calculated using PBE-D3­(BJ)^44,45^ method and a Γ-centered 3 × 3 × 1 k-point grid, while the total energies were refined using PBE-D4 ^46^ theoretical level, a 7 × 7 × 1 k-point grid, and the tetrahedron smearing method with Blöchl corrections.^47^ The electronic and ionic convergence criteria were set to 10^–5^ eV and 10^–4^ eV, respectively, and Gaussian smearing with a smearing width of 0.05 eV was used throughout the computations.

#### Surface Model

A three-layer 8 × 8 slab representing the Au(111) surface in DFT calculations was constructed using the experimental Au bulk lattice constant of 4.08 Å.^48^ To avoid interaction between the slabs, the cell was expanded to 25 Å in the nonperiodic *z*-direction by introducing a vacuum of *ca*. four times the slab height and a dipole correction^49^ along the *z*-direction was applied. During the structural optimizations the two bottom Au layers were fixed in their bulk positions, while the remaining atoms were allowed to relax. The adsorbates were initially placed approximately 3 Å above the Au(111) surface.

The adsorption energies (Δ*E*_ads_) were computed according to the expression:

$$\Delta E_{\mathrm{ads}}=E_{\mathrm{molecule}}{@}_{\mathrm{surface}}-\left(E_{\mathrm{molecule}}+E_{\mathrm{surface}}\right)$$

#### Adsorption Site Search

The most energetically preferred positions of the adsorbed species were determined by means of the working group’s own Global Minimum Adsorption Position Finder (GMAPF) algorithm that uses a Metropolis algorithm (5000 iterations) with simulated annealing at 5000 K followed by a molecular dynamics simulation, all using a cluster-based approach for the surface model and at the GFN1-xTB level of theory.^50^ The obtained structures were used as starting points in DFT calculations. A two-layer surface slab built according to above-mentioned procedure was aligned in the xy-plane and the maximum molecule–surface distance was set to 5 Å. Molecular dynamics simulations were performed in *NVT* ensemble at *T* = 300 K with a total runtime of 50 ps, a trajectory printout interval of 50 fs and a propagation step size of 4 fs. SCC accuracy level was set to 2.0 and hydrogen atom mass was adjusted to 4 times. Bond constraint was achieved using the SHAKE algorithm with an additional force constant constraint of 1.0 E_h_ a_0_^–2^ for the metal atoms.

#### Gas Phase Electronic Structures and dI/dV Simulations

The optimized geometries in the adsorbed (dome, bowl) and gas phases as described above are used for the electronic properties calculation using CP2K,^51^ employing PBE0 and Geodecker–Teter–Hutter (GTH) pseudopotentials^52^ and a TZV2P-MOLOPT-GTH basis set^53^ including D3­(BJ)^44,45^ dispersion corrections. To simulate constant height dI/dV maps using a fixed CO tip, PPSTM code^54^ is employed. For the relaxed scan simulation, PPAFM code^55^ was initially used to model the positions of the CO tips. The lateral stiffness for the CO tip was set to 0.25 N/m, and an oscillation amplitude of 1.0 Å was used. A broadening parameter of 0.1 eV was applied to all simulations. The CP2K spm tools python toolkit has been used to simulated STM images with p-wave character.^56^ The calculation results were analyzed with the help of Multiwfn package^57^ and visualized by Visual Molecular Dynamics.^58^

## Supplementary Material



## Notes

Experimental and computational details, additional STM and STS data, frequency shift versus tip–sample distance curves, simulated nc-AFM and BR-STM images, inversion experiments and calculated inversion barriers, computational adsorption geometries and molecular orbitals, precursor synthesis (PDF)
