# Supplementary material for: On-Surface Synthesis of a Nitrogen-Doped Curved Cycloarene: π‑Extended Pentaazaquintulene and Its Gold Complex
Source: J Am Chem Soc. 2025 Nov 11;147(47):43501–8. doi: 10.1021/jacs.5c11883 (PMC12673598; doi:10.1021/jacs.5c11883)
Supplement: Supplementary file 1 [file ja5c11883_si_001.pdf]

Supporting Information

On-Surface Synthesis of a Nitrogen-Doped Curved Cycloarene: π-Extended Pentaazaquintulene and its Gold Complex

Zilin Ruan[1], Olaf A. Kleykamp[1], Kiyan Linus Haiko Pohl [2,3], Tim Naumann[1], L. Alix Kaczmarek[2,3], Anton S. Nizovtsev[2,3,4,5], Eugen Sharikow[1], Jörg Sundermeyer[1], Doreen Mollenhauer[2,3,4,5,6], J. Michael Gottfried[1]

[1]*Philipps-Universität Marburg, Fachbereich Chemie, Hans-Meerwein-Str. 4, 35032 Marburg, Germany, gottfried@uni-marburg.de*

[2]*Justus Liebig Universität Gießen, Heinrich-Buff-Ring 17, 35392 Giessen, Germany*

[3]*Center for Materials Research (LaMa), Justus-Liebig University Giessen, 35392 Giessen, Germany*

[4]*Helmholtz-Institut für Polymere in Energieanwendungen, Lessingstr. 12–14, 07743 Jena, Germany*

[5]*Helmholtz-Zentrum Berlin für Materialien und Energie GmbH (HZB), Berlin, Germany*

[6]*Institut für Technische Chemie und Umweltchemie, Friedrich-Schiller-Universität Jena, Philosophenweg 7a, 07743 Jena, Germany*

1. Overview STM image after annealing to 480 K

Figure S1. Representative species on the surface after annealing to 480 K. (a) Large-scale and (inset) magnified STM images. The different Ullmann coupling products of the precursor 4-([1,1'-biphenyl]-2-yl)-2,6-dibromopyridine are denoted by numbers, referring to the structures in (b). (b) The corresponding chemical structures of the species in (a). Scanning parameters: (a) V_s = 0.15 V, I_t = 20 pA. Scale bar: (a) 7 nm; Inset: 2 nm.

2. Partially cyclodehydrogenated intermediates obtained at 620 K

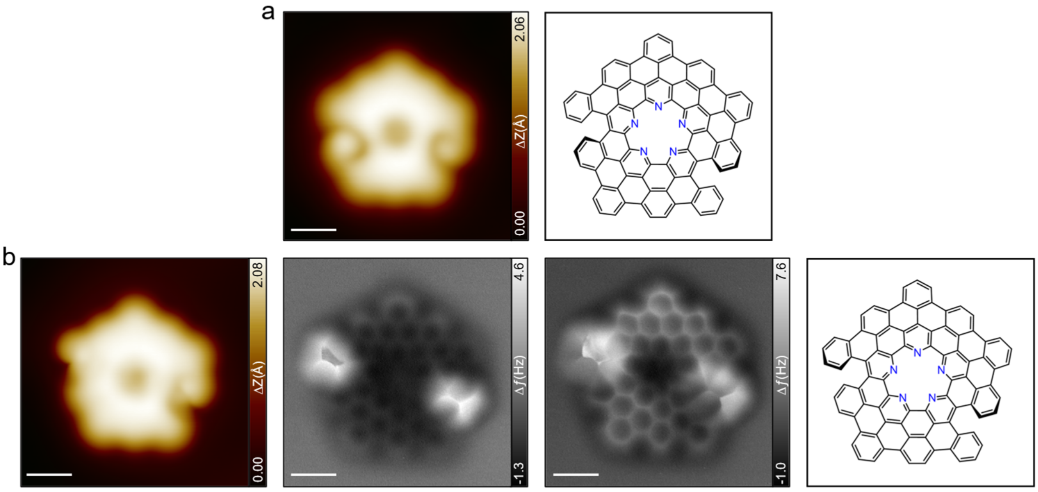

Figure S2. Characterization of intermediates obtained at 620 K. (a) (left) STM image of the partially cyclodehydrogenated intermediates and (right) its corresponding chemical structure. (b) (left) STM, (center) nc-AFM images acquired at different tip height and (right) the corresponding chemical structure of the intermediate. Scanning parameters: STM, (a) V_s = 0.15 V, I_t = 20 pA; (b) V_s = 0.1 V, I_t = 50 pA; nc-AFM, V_s = 10 mV. Scale bar: STM, (a, b) 0.6 nm; nc-AFM, 0.5 nm.

3. Partially cyclodehydrogenated intermediates obtained at 630 K

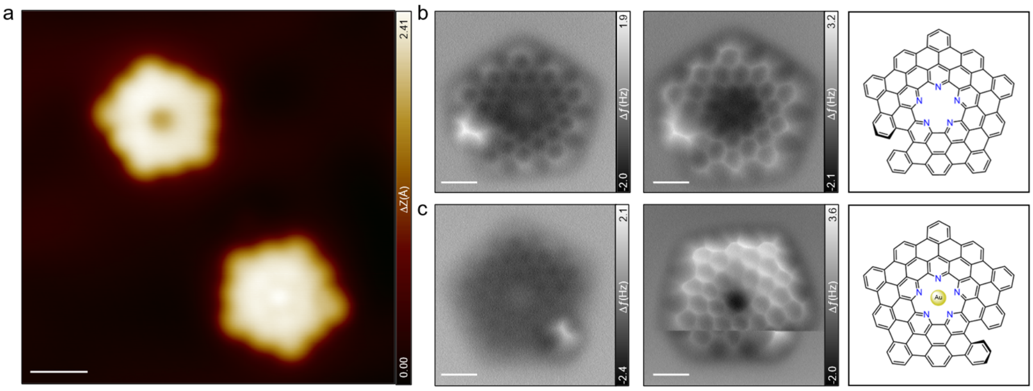

Figure S3. Characterization of intermediates obtained at 630 K. (a) STM image of partially cyclodehydrogenated intermediates with empty and filled cavity. (b, c) (left to right) CH nc-AFM images obtained at different tip height and the corresponding chemical structures. Scanning parameters: (a) V_s = 0.2 V, I_t = 20 pA; (b, c) V_s = 5 mV. Scale bar: (a) 1.2 nm; (b, c) 0.5 nm.

4. High-resolution images of defective pentamers

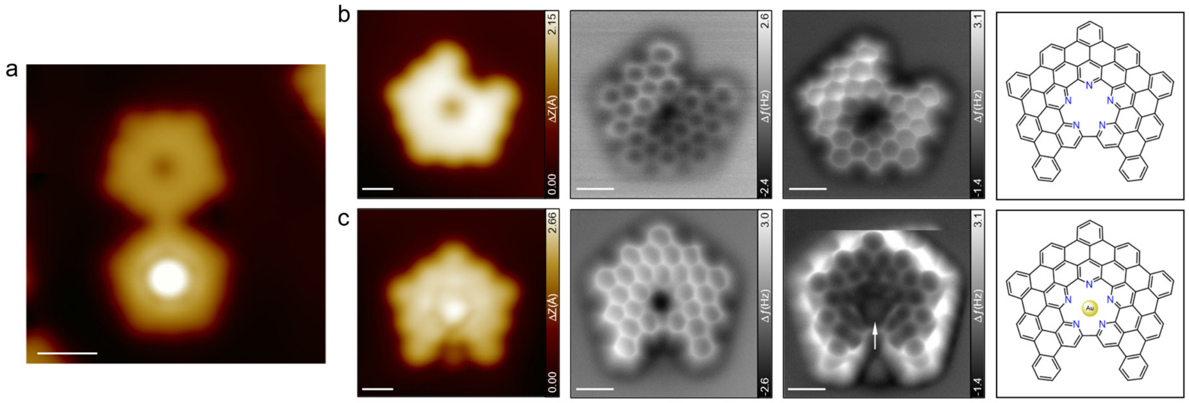

Figure S4. Characterization of defective pentamers with empty and filled cavity. (a) STM images of two coupled pentameric species. (b) (left to right) STM, CH-nc-AFM images with different tip-sample separation and the corresponding chemical structure of a defective pentamer with empty cavity, a phenyl group is lost after annealing. (c) (left to right) STM, CH-nc-AFM, CC-nc-AFM images and the corresponding chemical structure of a defective pentamer as shown in (b) but with Au-filled cavity. The gold adatom is denoted by the white arrow. Scanning parameters: (a) STM, V_s = 0.15 V, I_t = 50 pA; nc-AFM: V_s= 10 mV. Scale bar: (a) 1 nm; (b, c) 0.5 nm for all images.

5. Additional high-resolution images of the extended N-doped quintulene with and without a gold adatom inside its cavity

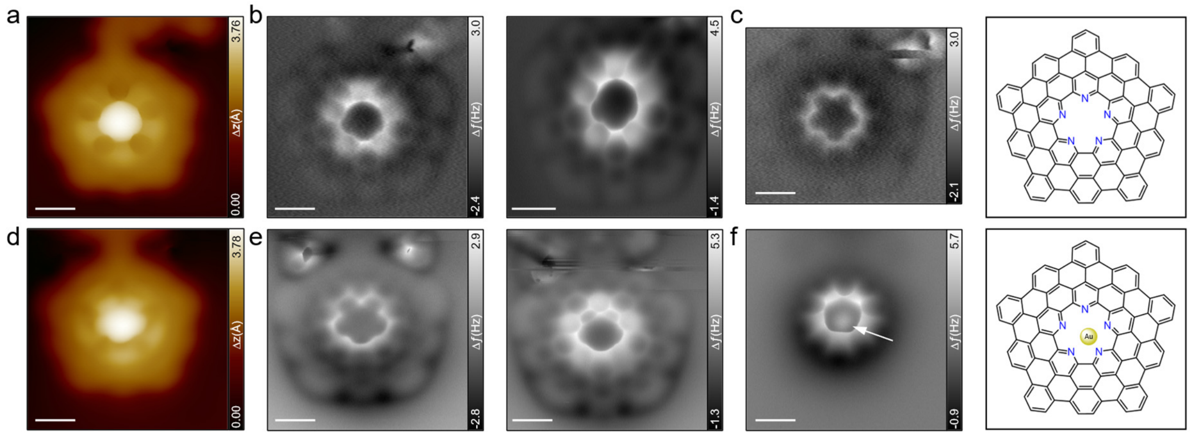

Figure S5. Additional high-resolution images of the π-extended pentaazaquintulene its Au complex. (a-c) Adsorbed π-extended pentaazaquintulene and (d-f) its gold complex(a, d) STM, (b, e) CC nc-AFM images at different tip heights, and (c, f) (left) CH nc-AFM images and (right) the corresponding chemical structures of the two species. The arrow in (f) highlights the coordinated Au adatom. Scanning parameters: (a, d) V_s = 0.1 V, I_t = 50 pA; (b, c, e, f) V_s = 10 mV. Scale bar: (a, d) 0.6 nm; (b, c, e, f) 0.5 nm.

6. Frequency shift versus tip-sample distance curves for π-extended pentaazaquintulene-Au complex

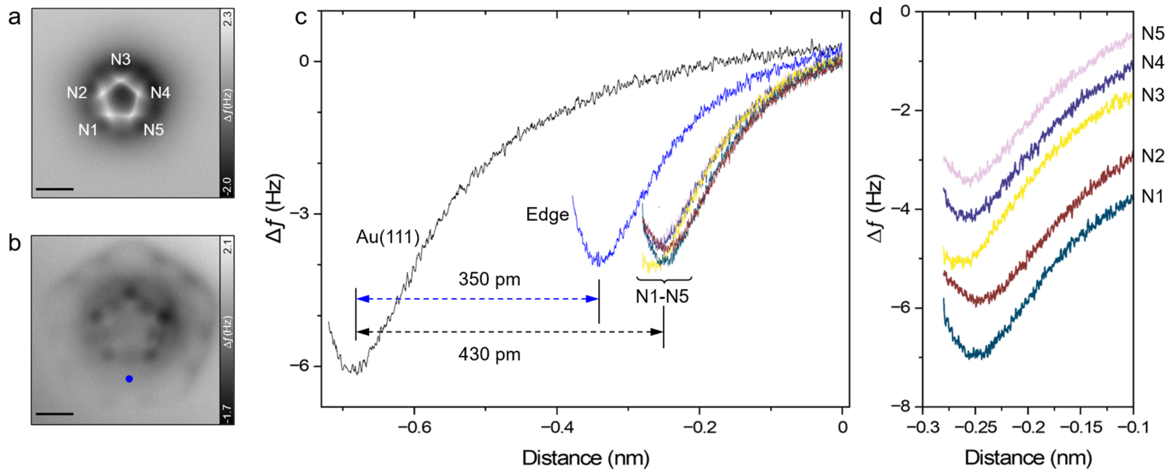

Figure S6. Adsorption height of the pentaazaquintulene-Au complex. (a) CH-nc-AFM image of the complex, same as show in Figure 3g, the five nitrogen atoms are denoted by N1-N5, respectively. (b) CC-nc-AFM image of the same molecule as shown in (a). (c) Frequency shift versus tip-sample separation acquired at positions marked in (a, b). (d) Magnified view of the curves shown in (c).

7. Simulated nc-AFM images of the dome-shaped structure

We simulate the constant height nc-AFM image employing the DFT equilibrium dome-shaped structure, and good agreement is achieved. The slightly different height of the nitrogen atoms in the complex structure is not captured in our nc-AFM simulation (not shown), we attribute this variation to the charge redistribution and possibly a slight local deformation of its geometry induced by the tip.

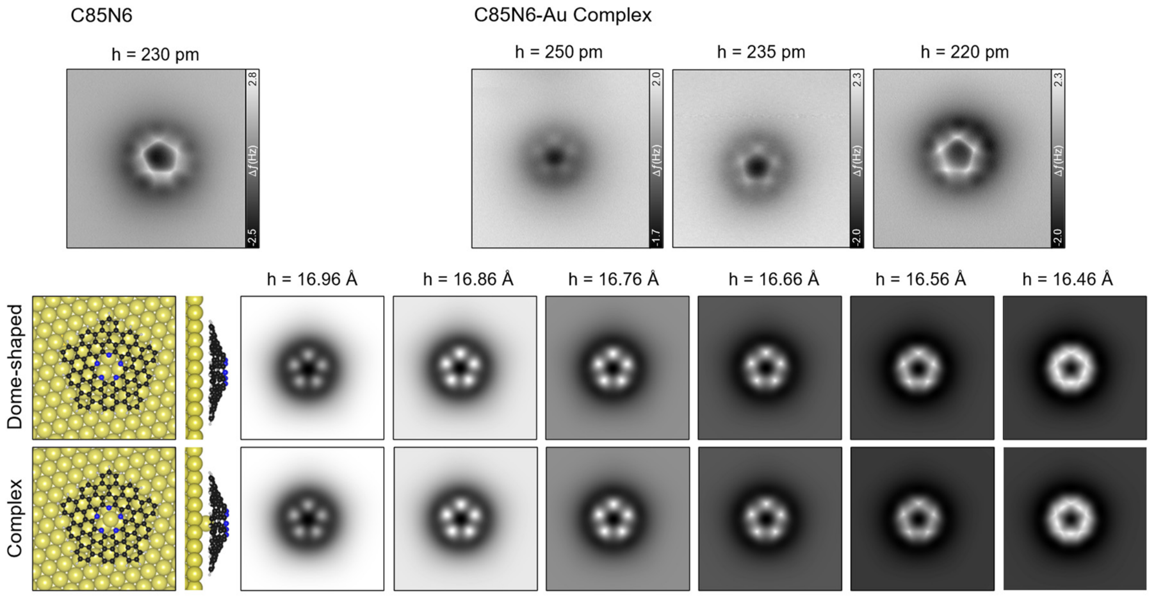

Figure S7. Simulated nc-AFM images for the C85N5 and its gold complex. The indicated tip heights are with respect to the setpoint V_s = 10 mV, I_t = 5pA (0 pm) at which the feedback loop is switched off. Images size: 2.5 nm × 2.5nm.

8. BR-STM simulation of the π-extended pentaazaquintulene

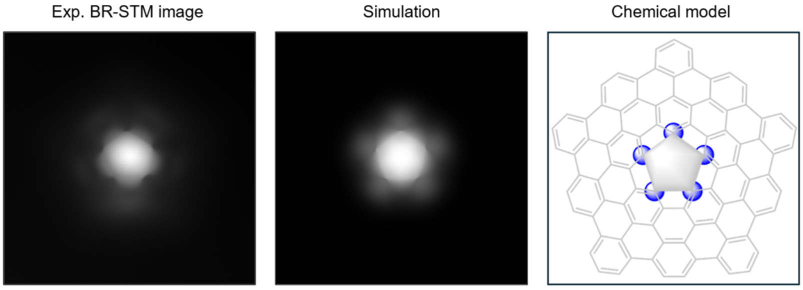

Figure S8. Comparison of experimental and simulated BR-STM image.

9. Convex to concave inversion induced by STM tip

The extended N-doped quintulene with empty cavity can be inverted by the STM tip, as indicated by the white arrows in Figure S9. Specifically, the tip is positioned over the cavity, and then the bias was ramped from 0.5 V to around 2.2 V, an abrupt change in the tunneling current indicates an inversion event. In contrast to this manipulation, this inversion cannot be achieved for the corresponding Au complex, possibly due to high repulsion.

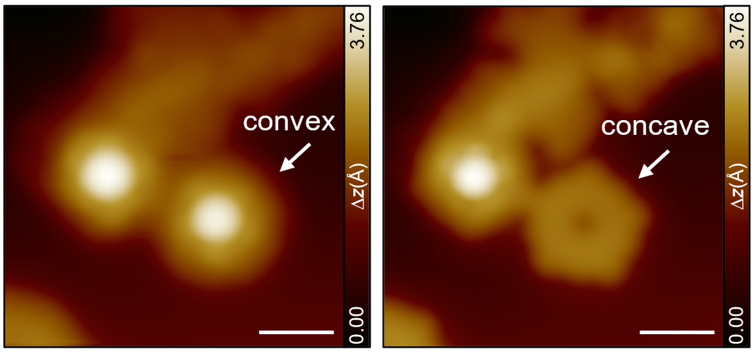

Figure S9. Tip-induced conversion of the convex to the concave form of the extended N-doped quintulene. The convex structure of the nanocone (left) is converted to the concave structure (right) by the STM tip manipulation. Scanning parameters: V_s = 0.15 V, I_t = 20 pA. Scale bar: 1.2 nm.

10. Increased yield of bowl-shaped 3 at higher annealing temperature

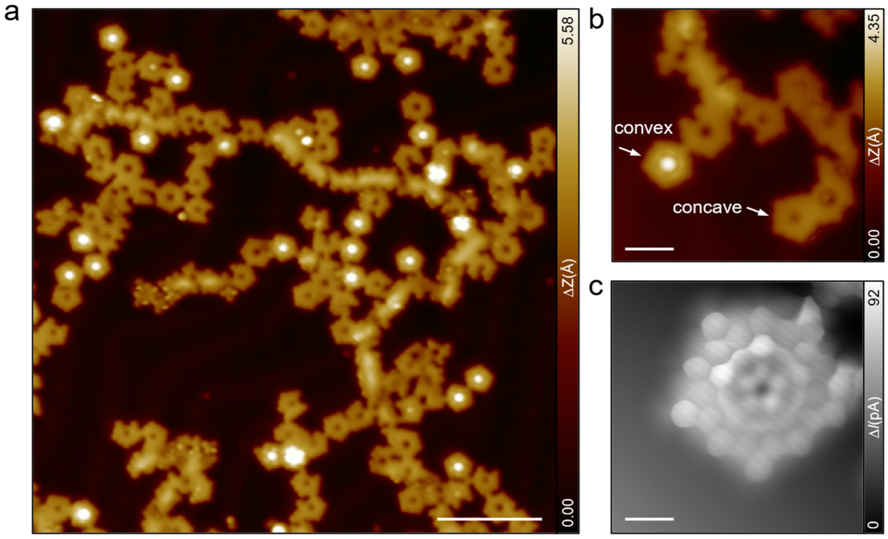

Figure S10. Formation of nanocones at higher temperature. (a) Overview STM image after further annealing of the sample to 670 K. (b) STM image showing the coexistence of the convex and concave structures, as indicated by arrows. (c) Zoom-in high-resolution BR-STM image of the concave nanocone in (b). Scanning parameters: (a, b) V_s = 0.3 V, I_t = 20 pA; (c) V_s = 5 mV. Scale bar: (a) 7 nm; (b) 2 nm; (c) 0.6 nm.

11. Frequency shift versus tip-sample distance curves for the bowl-shaped structure

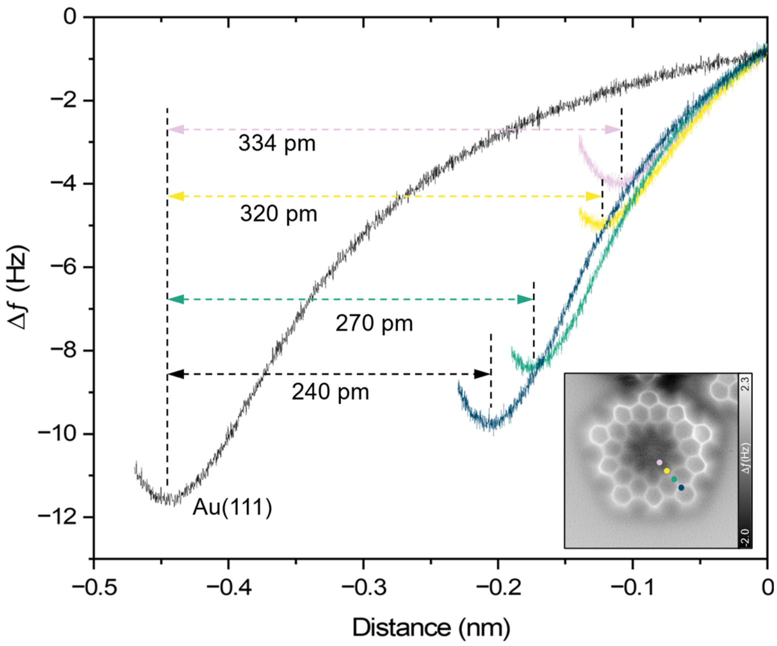

Figure S11. Adsorption height of bowl-shaped 3. The curves are measured at spots indicated in the inset.

12. nc-AFM simulation for the bowl-shaped structure

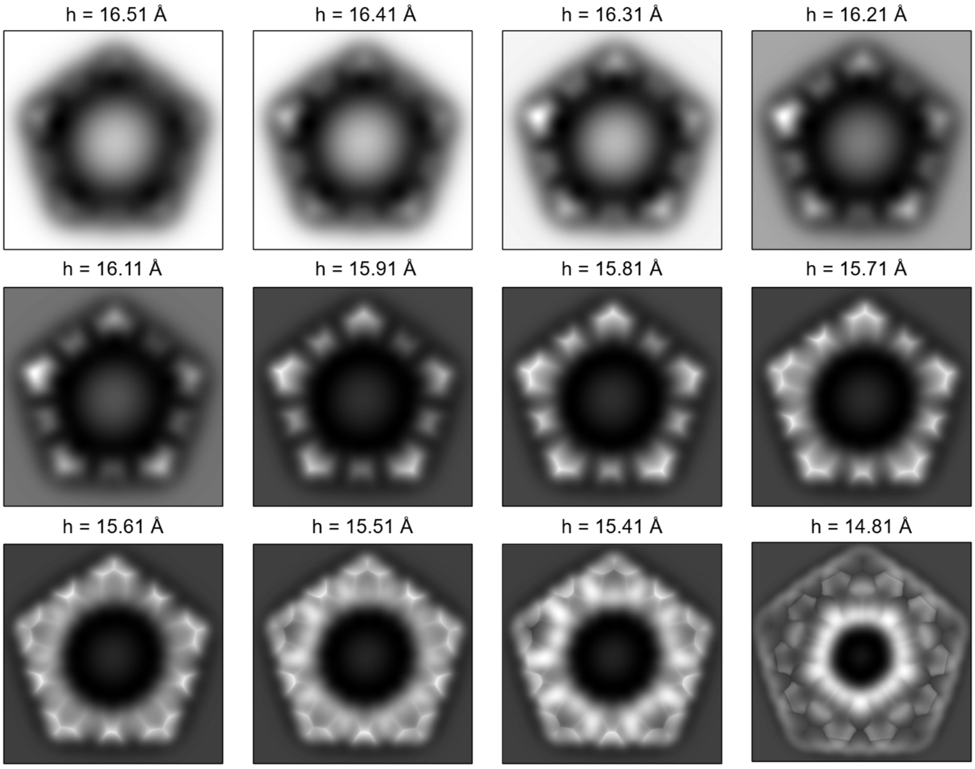

Figure S12. Simulated varied height nc-AFM images for the bowl-shaped structure. Note that the experimental features are not fully captured by the simulated nc-AFM images based on the DFT equilibrium geometry, likely due to the known tendency of Grimme D3 dispersion corrections to overestimate adsorption heights[1,2].

13. Calculated bowl-to-dome inversion barrier

Non-periodic gas-phase DFT calculations

A conformer search for N-doped quintulene was performed using semiempirical GFN2-xTB method[3] implemented into the extended tight binding program package (xTB 6.3.3)[4] and coupled with the conformer-rotamer ensemble sampling tool (CREST 2.12)[5]. The structures of the five most energetically stable conformers were reoptimized using B3LYP hybrid density functional[6-8] combined with def2-TZVP basis set[9,10] and the resolution of identity (RI) approximation employing Turbomole Version 7.5[11,12]. The lowest-energy conformer obtained was used in further calculations.

The inversion barrier was computed at the B3LYP/def2-TZVP level of theory using ORCA 6.0 program package[13]. All stationary points were characterized as minima (the number of imaginary frequencies (NImag) was equal to zero) or saddle points (NImag > 0) by a vibrational analysis. A direct connection of a transition state found with bowl and dome conformations of N-doped quintulene was confirmed by the intrinsic reaction coordinate (IRC)[14] calculations. In these DFT computations the RIJCOSX approximation[15,16] with the corresponding auxiliary basis sets[10] was employed to accelerate convergence and DefGrid2 integration grid was used.

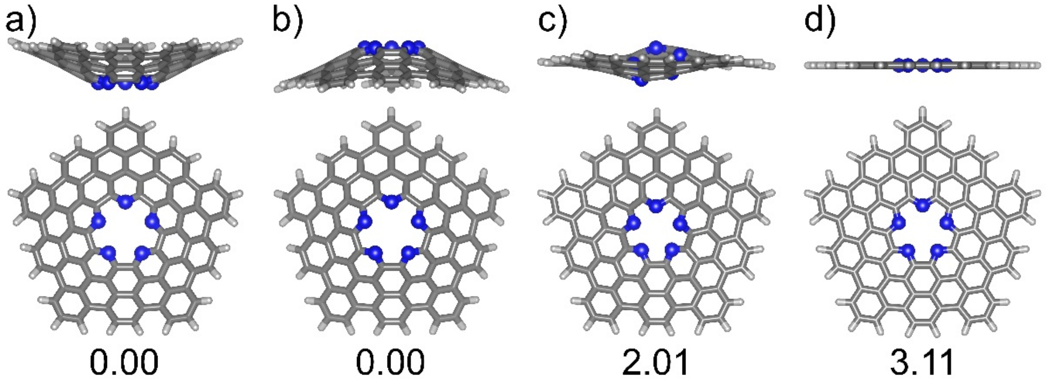

Figure S13. Top and side views of N-doped quintulene in its bowl (a) and dome (b) conformations and curved (c; NImag = 1) and flat (d; NImag = 3) transition state structures for the bowl-to-dome inversion calculated at the B3LYP/def2-TZVP level of theory. Computed zero-point vibrational energy corrected inversion barriers ($\Delta H^{\neq}_{0\,K}$) are given in eV. Color code: H (light gray), C (gray), N (blue).

14. Energy decomposition analysis

Table S1. Decomposition of the adsorption energy (ΔE_{ads}) calculated by PBE-D4 method into DFT (E_{DFT}) and dispersion (E_{disp}) parts. All values are given in eV.

Species	E_{DFT}	E_{disp}	ΔE_{ads}
bowl@Au(111)	0.820	-2.401	-1.581
dome@Au(111)	1.085	-2.772	-1.687
dome@Au@Au(111)	0.416	-2.542	-2.127

15. N-Au in-plane distances for the N-doped quintulene-Au complex

Table S2. In-plane distances (d, Å) between the nitrogen atoms and the gold adatom of adsorbed N-doped quintulene-Au complex calculated by PBE-D3(BJ) method.

Atom	d, Å
N1	2.048
N2	1.866
N3	2.275
N4	2.632
N5	2.514

16. Closest nitrogen-nitrogen distances

Table S3. Distances (d, Å) between the closest nitrogen atoms of adsorbed N-doped quintulene calculated by PBE-D3(BJ) method.[a,b]

Atoms	d, Å		
	bowl	dome	complex
N1–N2	2.630	2.635	2.644
N2–N3	**2.580**	2.635	2.642
N3–N4	**2.597**	2.633	2.644
N4–N5	**2.614**	2.636	2.646
N5–N1	2.627	2.635	2.644

[a] Calculated N–N distance for the molecule in *vacuo* is 2.653 Å.

[b] Distances with large deviation from the mean value are shown in bold.

17. Adsorption geometry of the π-extended pentaazaquintulene and its gold complex

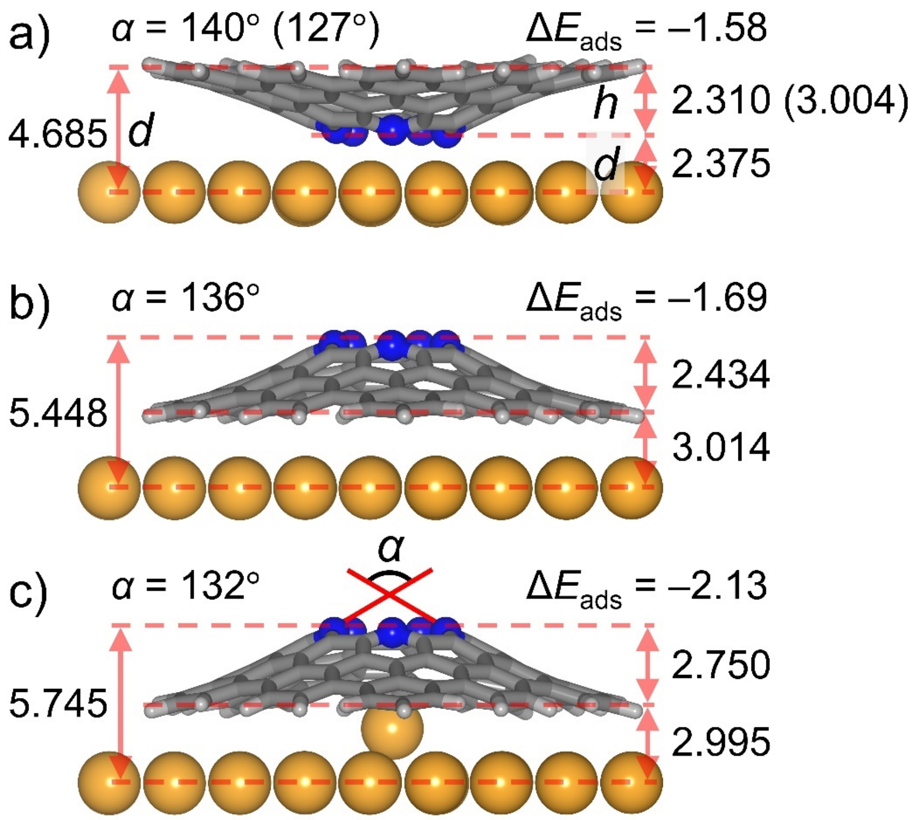

Figure S14. The most relevant structural parameters of N-doped quintulene in its bowl conformation adsorbed on the Au(111) surface, same as shown in Figure 3, here shown again for clarity. (a) and dome conformation adsorbed on the Au(111) (b) and Au(111) with Au adatom (c) surfaces are given according to PBE-D3(BJ) calculations. Average adsorption distances of non-hydrogen atoms located closest and farthest from the Au(111) surface (d, Å), depths of molecules (h, Å), and bending angles (α, degrees) are presented. Values of h and α calculated for molecule in *vacuo* are given in parentheses. Computed adsorption energies (ΔE_{ads}, eV) are also shown. Color code: H (light gray), C (gray), N (blue), Au (golden).

18. Additional dI/dV curves for the π-extended pentaazaquintulene and calculated DOS

The peak at around 1 V, is more pronounced when the tip approaches the cavity (Figure S15a and b). We attribute these peaks to the confined states of the adsorption of the molecule to the surface rather than molecular orbitals related resonance, as justified by the calculated density of state near Fermi energy and the corresponding molecular orbitals (Supplementary Figures S16-S18 and S21).

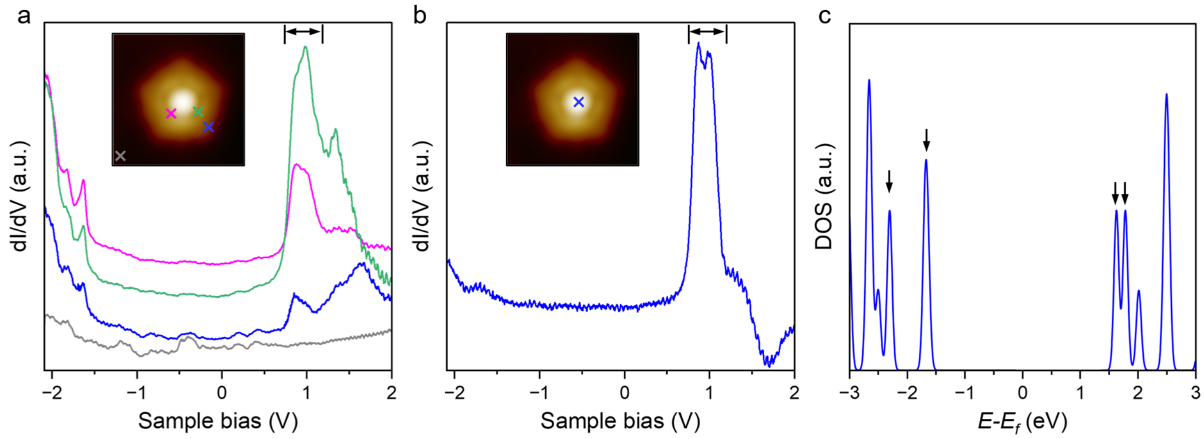

Figure S15. Additional STS spectra and calculated DOS for the dome-shaped π-extended pentaazaquintulene in the gas phase. Arrows in a) and b) mark the peaks corresponding to the surface states and arrows in c) mark orbitals accessed in the experiment.

19. Calculated projected density of states of adsorbed system.

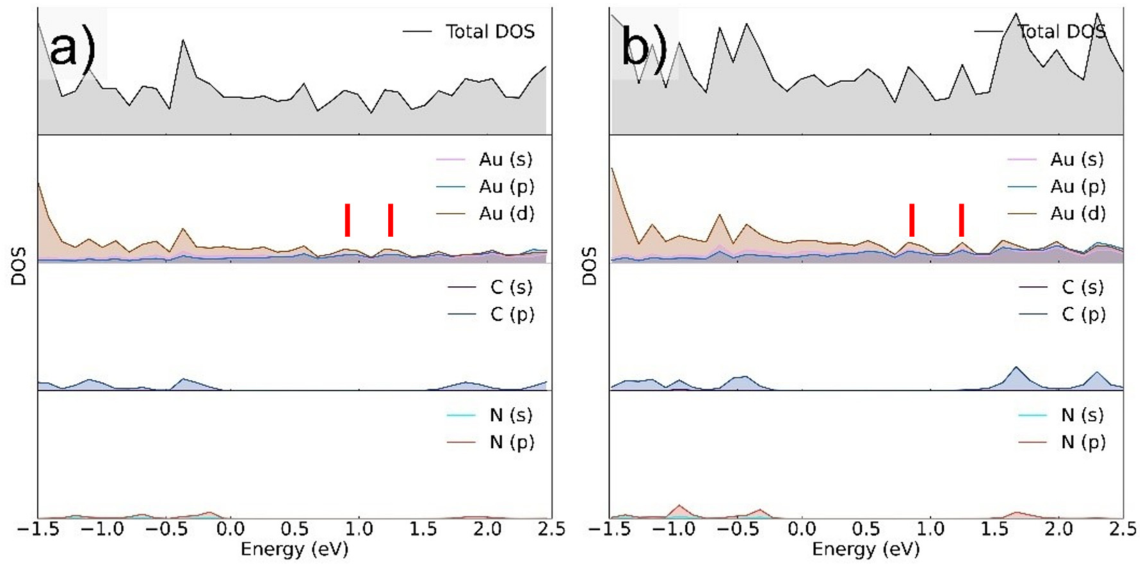

Figure S16. Calculated DOS of C85N5 and its gold complex adsorbed on Au(111). Total and projected DOS of adsorbed π-extended pentaazaquintulene (a) and its Au complex (b) calculated by PBE-D4 method under periodic boundary conditions. The confined states are indicated by red lines.

20. Constant current orbital/LDOS simulations.

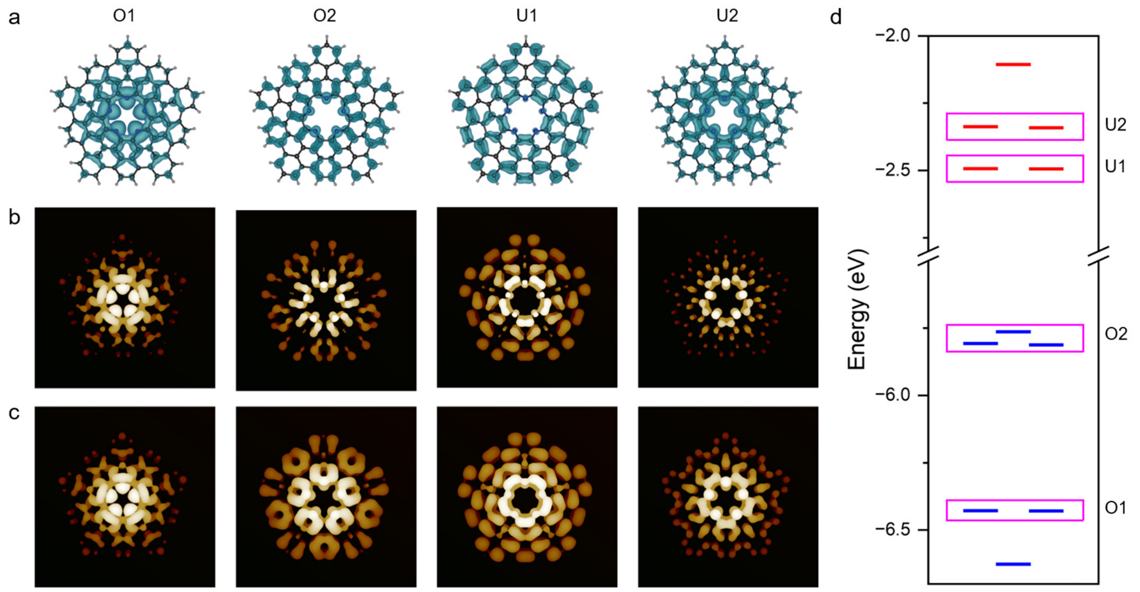

Figure S17. **Calculated orbital density and LDOS maps.** (a) Calculated orbital density maps and LDOS maps. (b) Maps obtained with larger isovalues. (c) Maps obtained with smaller isovalues. (d) Experimental dI/dV maps (as shown in Figure 4 of the main text). (e) Orbitals considered in the calculations.

21. Comparison of experimental and simulated STM images

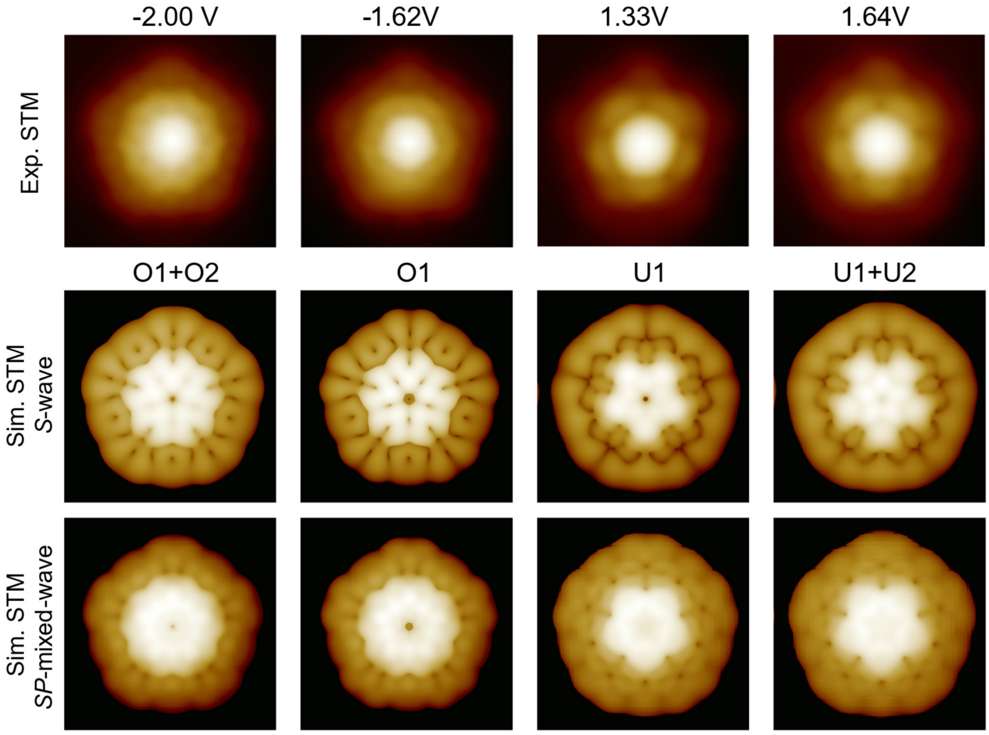

Figure S18. **Comparison of the experimental STM images taken at energies at the peaks R1-R4 (Figure 1) and the simulated STM images of a gas phase C85N5 considering a s-wave and sp-mixed-wave tip (13%s+87%p).**

22. dI/dV map simulation using tips terminated with different wavefunction

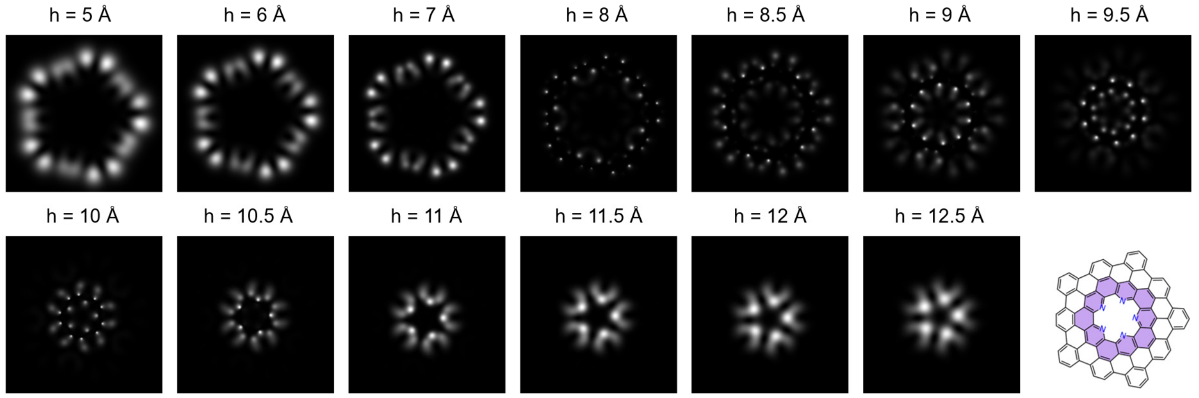

Figure S19. Simulated dI/dV maps of the HOMO using an *s*-wave terminated tip. We note that the simulated local density of state (LDOS) maps do not align well with the experimental dI/dV maps, which we attribute to the deformation of the molecule caused by the CO-decorated tip.

23. dI/dV map simulation using tips terminated with different wavefunction

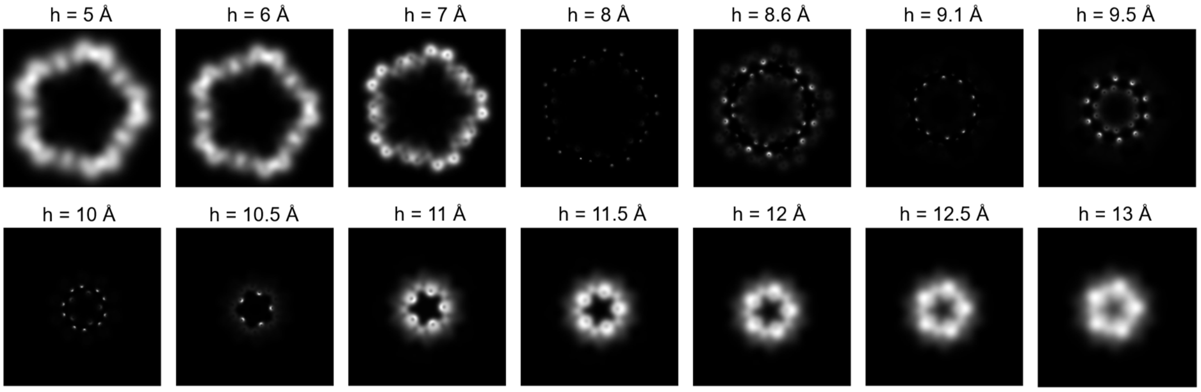

Figure S20. Simulated dI/dV maps of the HOMO using a CO-terminated tip with 87% *s*- and 13% *p*-wave character.

24. dI/dV map simulation using tips terminated with different wavefunction

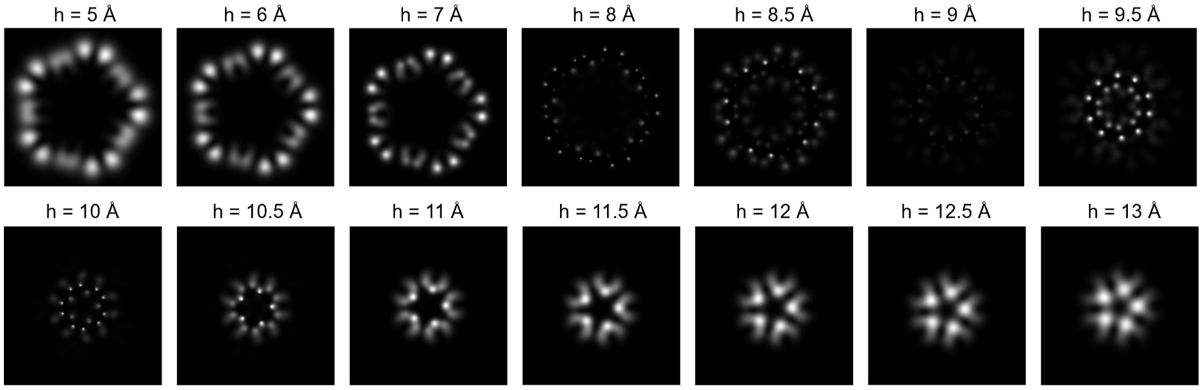

Figure S21. Simulated dI/dV maps of the HOMO using a CO-terminated tip, with 75% *s*- and 25% *p*-wave character.

25. STS measurement of the N-doped quintulene-Au complex

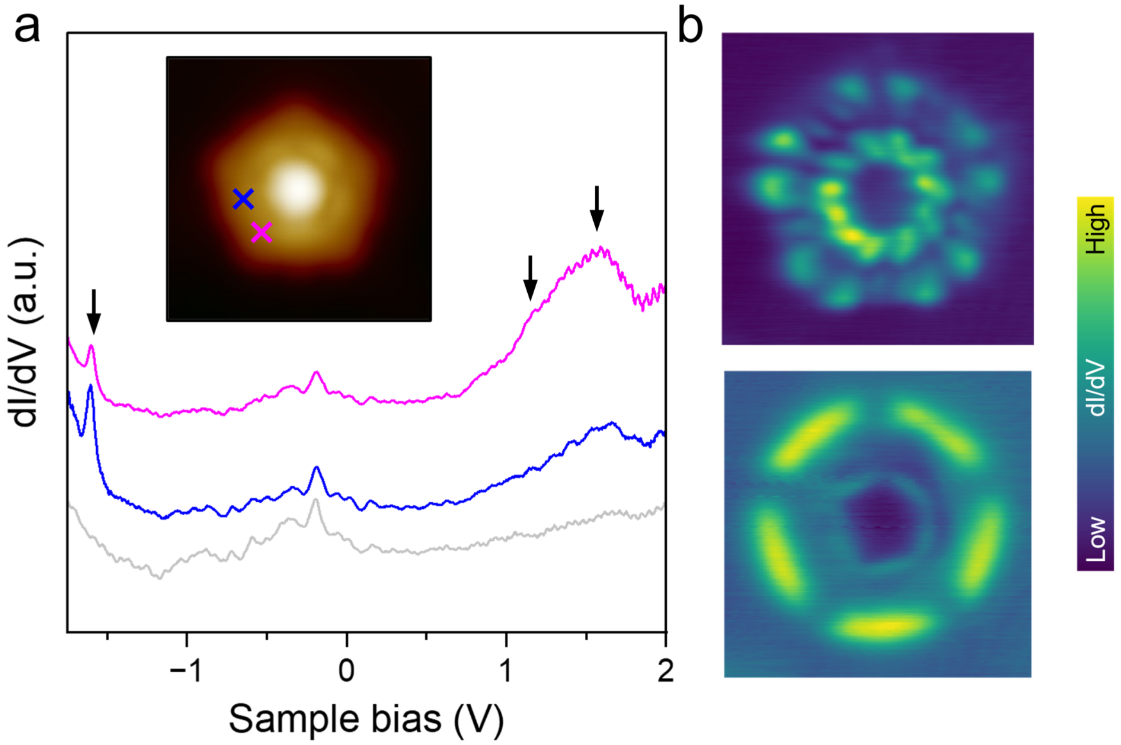

Figure S22. Characterization of the extended N-doped quintulene-Au complex. (a) dI/dV curves measured at spots indicated in the inset. (b) dI/dV map taken at HOMO (-1.59 V, top) and LUMO+1 (1.59 V, bottom).

26. Calculated molecular orbitals for the gas phase structure

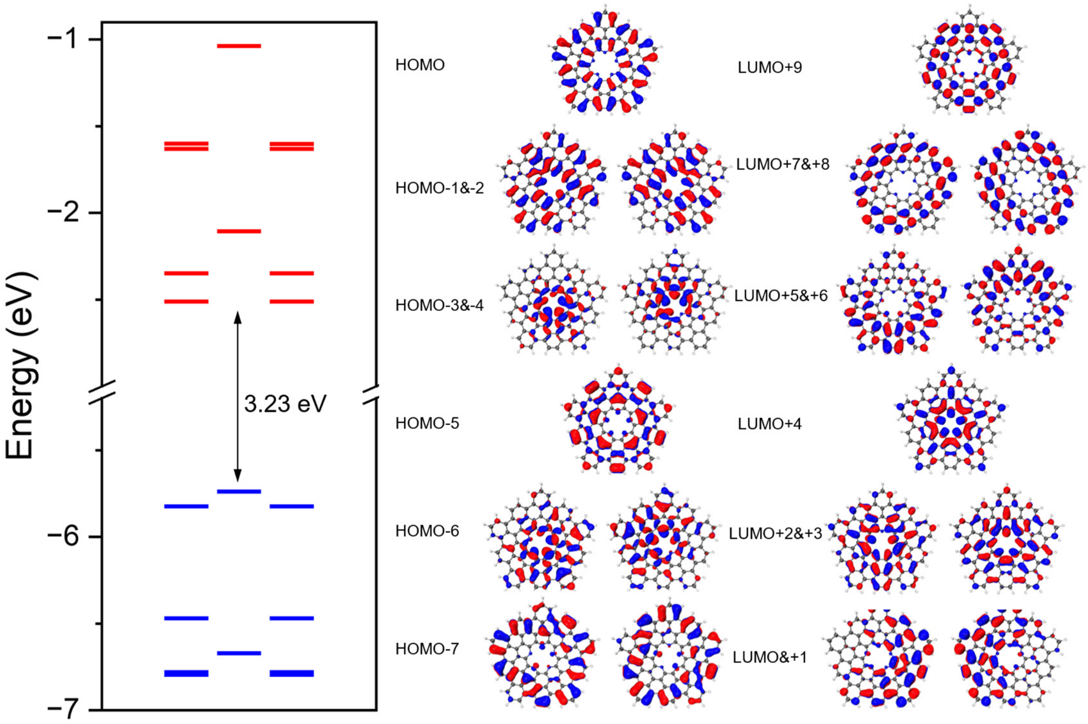

Figure S23. Molecular orbitals of N-doped quintulene (gas phase structure) calculated by PBE0-D3(BJ) method.

27. Calculated molecular orbitals for the dome-shaped structure

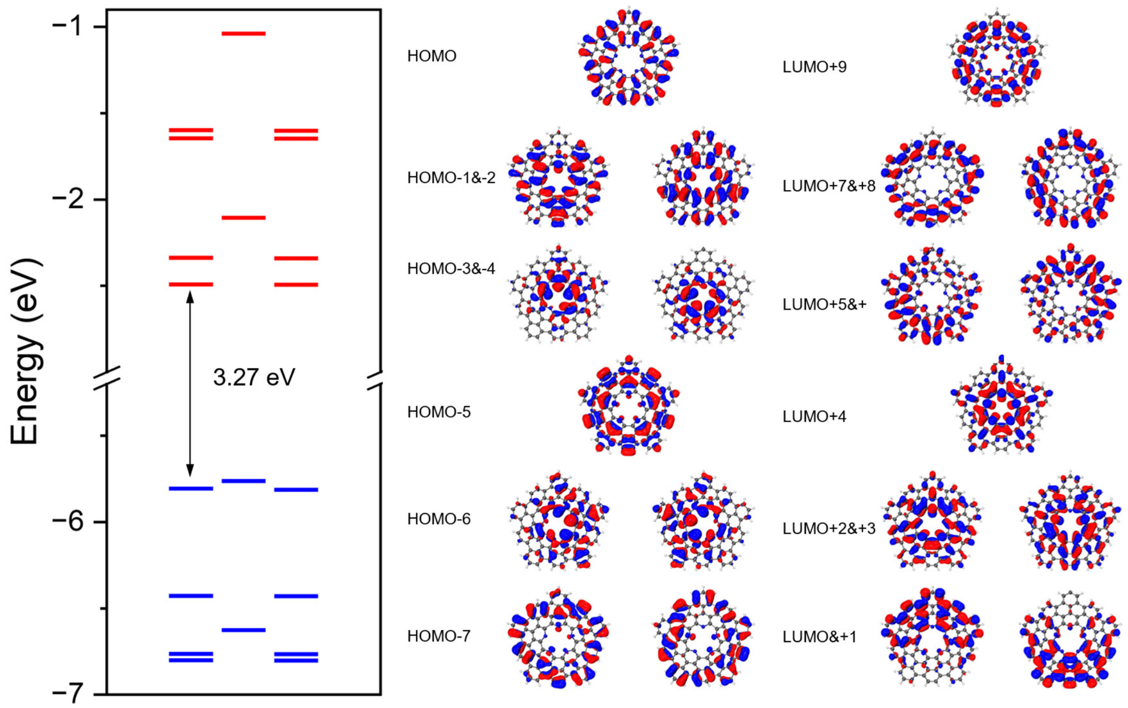

Figure S24. Molecular orbitals of N-doped quintulene (dome-shaped structure) calculated by PBE0-D3(BJ) method.

28. Calculated molecular orbitals for the bowl-shaped structure

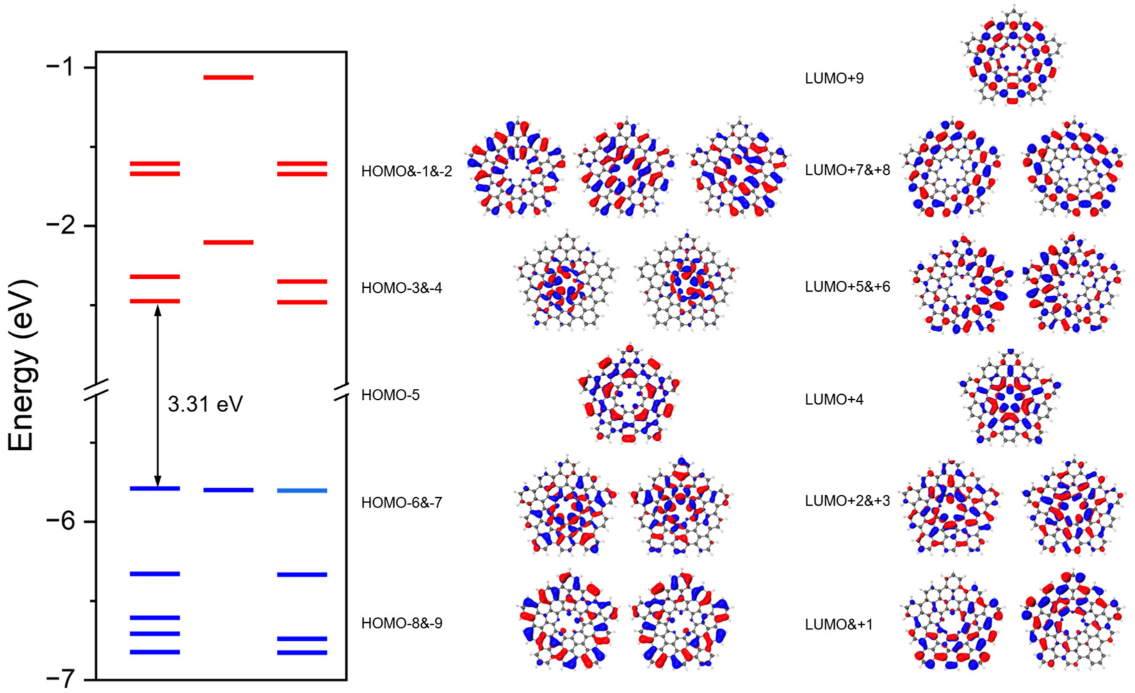

Figure S25. Molecular orbitals of N-doped quintulene (bowl-shaped structure) calculated by PBE0-D3(BJ) method.

29. Materials, Methods and Devices

All reactions were carried out under inert atmosphere using standard SCHLENK techniques. Air or moisture sensitive substances were stored in a nitrogen flushed glovebox. Solvents were purified according to common literature procedures and stored under an inert atmosphere over molecular sieves (3 Å or 4 Å)[17]. For thin-layer chromatography, TLC plates from Merck KGaA with silica gel 60 on aluminum with fluorescence-quenching F254 at room temperature were used. ^{1}H and ^{13}C NMR spectra were recorded on Bruker Avance II 300, Avance III HD 300 or Avance III HD 500 spectrometers. Chemical shift δ is denoted relatively to $SiMe_4$ for ^{1}H and ^{13}C. ^{1}H and ^{13}C NMR spectra were referenced to the solvent signals. Multiplicity is abbreviated as followed: s (singlet), d (doublet), t (triplet), q (quartet), m (multiplet), br (broad signal). HR-ESI mass spectra were acquired with an Orbitrap Q Exactive plus mass spectrometer (Thermo Fischer Scientific). The resolution was set to 140.000. Melting point was measured using a Büchi B-540 melting point apparatus with a heating rate of 0.2 °C/min. The last solvent used is shown in parentheses.

The data collection for the single-crystal structure determination was performed on a Bruker D8 Quest diffractometer by the X-ray service of Fachbereich Chemie, Universität Marburg. Information concerning the used hardware, and software used for data collection, cell refinement and data reduction as well as structure refinement can be reviewed in the electronic supplement tables and CCDC 2447190. The structure was solved using (SHELXT)[18] and refinement process (SHELXL 2019/1)[19] the data were validated by using Platon.[20] All graphic representations were created with Diamond 4.[21]

30. Synthesis

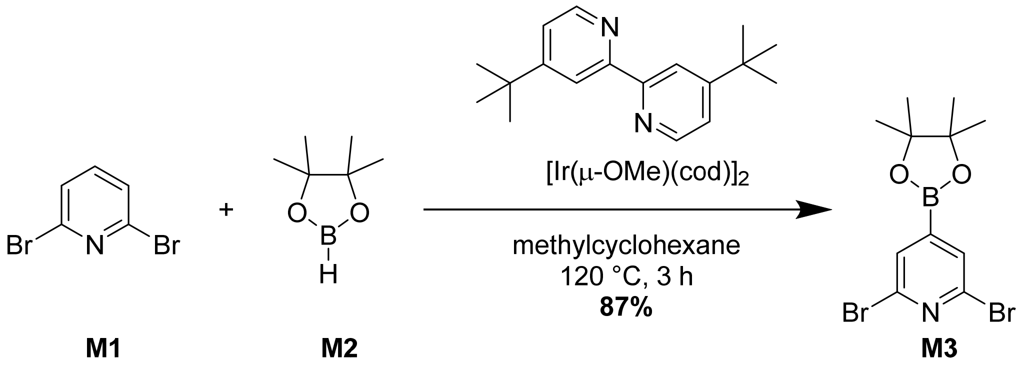

Under argon atmosphere, $[Ir(\mu\text{-}OMe)(cod)]_2$ (279 mg, 0.42 mmol, 0.02 eq.) and 4,4'-di-tert-butyl-2,2'-bipyridine (226 mg, 0.84 mmol, 0.04 eq.) were dissolved in dry methyl cyclohexane (75 ml). 2,6-Dibromopyridine (M**1**) (5.00 g, 21.1 mmol, 1.00 eq.) and 4,4,5,5-tetramethyl-1,3,2-dioxaborolane (M**2**) (6.13 ml, 42.2 mmol, 2.00 eq.) were added, subsequently the reaction mixture turned from yellow to dark red. It was stirred at 120 °C for 3 h. After consumption of the starting material monitored by TLC, the mixture was filtered through a pad of silica gel. The resulting oil was absorbed on silica gel and the crude product was eluted from the column using ethyl acetate. The solvent was removed to obtain the product M**3** as a white solid (6.68 g, 18.4 mmol, 87%).

The analytical data were in accordance with the literature:[22]

^{1}H NMR (300 MHz, CD_2Cl_2): δ (ppm): 7.76 (s, 2H, $H_{arom.}$), 1.33 (s, 12H, CH_3).
$^{13}C\{^1H\}$ NMR (75 MHz, CD_2Cl_2): δ (ppm): 141.0, 132.3, 85.7, 83.4, 25.0.
HR-MS (ESI$^+$) *m/z* calcd. for $[M+H]^+$ $C_{11}H_{14}BBr_2NO_2H$: 363.9539; found: 363.9514.

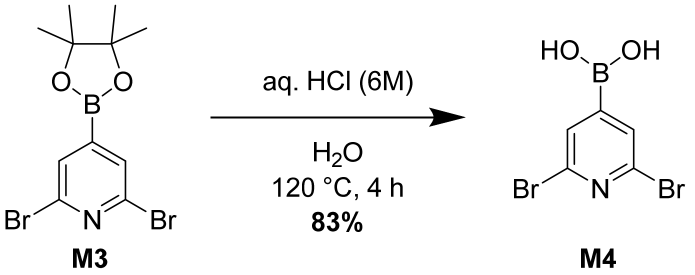

Boronic ester M**3** (2.00 g, 5.51 mmol, 1.00 eq.) was added to an aqueous HCl solution (6 M, 50 ml) and the mixture was heated to reflux for 4 h. When completion of the reaction was determined by TLC, the reaction mixture was cooled down to room temperature and ethyl acetate (50 ml) was added. The phases were separated, and the aqueous phase was extracted with ethyl acetate (3 x 20 ml). The combined organic phases were dried over Na_2SO_4, filtered and were concentrated *in vacuo* to obtain **M4** as an off-white solid (1.29 g, 5.51 mmol, 83%) which was used without further purification.

HR-MS (ESI$^+$) *m/z* calcd. for $[M+H]^+$ $C_5H_4BBr_2NO_2H$: 281.8755; found: 281.8745.

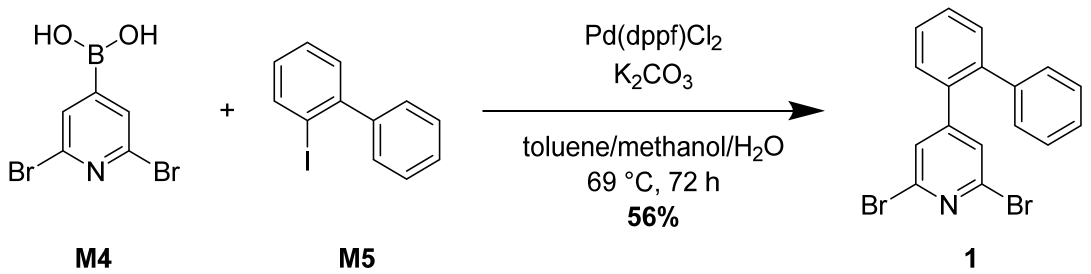

A Schlenk tube was charged with Pd(dppf)Cl_2 (234 mg, 0.32 mmol, 0.10 eq.) and K_2CO_3 (1.33 g, 9.62 mmol, 3.00 eq.) under argon atmosphere and degassed toluene (6 ml) was added. 2-Iodo-1,1'-biphenyl (M**5**) (0.87 ml, 4.81 mmol, 1.50 eq.) and (2,6-dibromopyridin-4-yl)boronic acid (M**4**) (0.90 g, 3.21 mmol, 1.00 eq.) were added. After addition of degassed methanol (2 ml) and degassed water (2 ml), the reaction was stirred at 69 °C for 72 h. The reaction mixture was filtered through a pad of celite, water (10 ml) was added to the filtrate, and the phases were separated. The aqueous phase was extracted with ethyl acetate (4 x 10 ml). The combined organic phases were dried over Na_2SO_4, the solvent was removed, and the crude product was purified *via* column chromatography (hexane/ethyl acetate 70:1) to obtain the target molecule **1** (0.70 g, 1.80 ml, 56%) as a white solid. For the purpose of UHV on surface coupling chemistry, the product was recrystallized from methylene chloride/*n*-hexane as

colorless in part single crystalline blocks (m.p.: 132.2 °C).

^{1}H NMR (500 MHz, $C_2D_2Cl_4$): δ (ppm): 7.55 – 7.50 (m, 1H, H_{arom}), 7.49 – 7.44 (m, 2H, H_{arom}), 7.38 (dd, J = 7.8, 1.4 Hz, 1H, H_{arom}), 7.31 (m, 3H, H_{arom}), 7.20 (s, 1.7H, H_{arom}), 7.11 (m, 2H, H_{arom}), 7.03 (m, 0.3H, H_{arom}).
^{13}C{^{1}H} NMR (125 MHz, $C_2D_2Cl_4$): δ (ppm): 154.6, 154.2, 149.7, 140.5, 139.9, 139.8, 139.3, 134.6, 134.5, 130.8, 129.7, 129.5, 128.3, 127.9, 127.7, 127.4, 123.9.
HR-MS (ESI^{+}) *m/z* calcd. for $[M+Na]^{+}$ $C_{17}H_{11}Br_2NNa$: 411.9130; found: 411.9121.
m.p. (methylene chloride): 132.2 °C

Note: Due to the ortho substitution pattern of the central phenyl ring and molecular dynamics of the precursor **1**, the two protons of the pyridine moiety separate in two signals at δ = 7.20 ppm and 7.03 ppm in the ^{1}H NMR spectrum. The same can be seen in the ^{13}C NMR where more signals than expected can be observed.

31. Single crystal X-ray structure

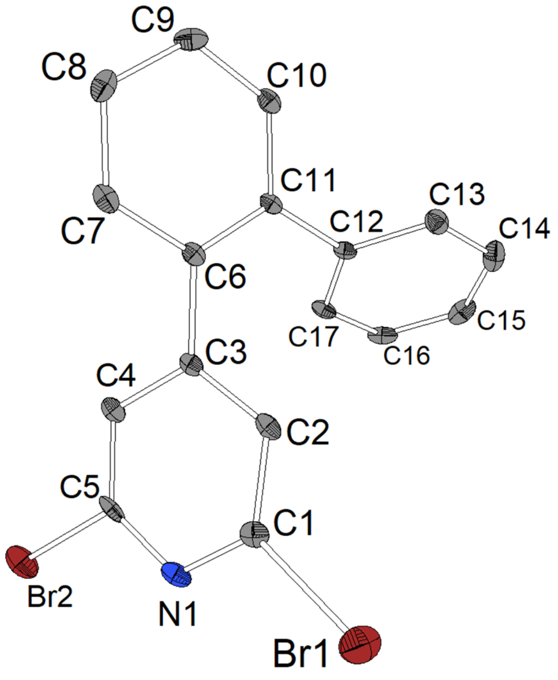

Figure S26. Solid state structure of **1**. Hydrogen atoms are omitted for clarity and thermal ellipsoids are shown at 50% probability level.

Table S2: Single crystal data of 1.

Identification code	**1**
CCDC code	2447190
Empirical formula	C17 H11 Br2 N
Formula weight	389.09
Temperature	100(2) K
Wavelength	0.71073 Å
Crystal system	Triclinic
Space group	P −1
Unit cell dimensions	a = 7.7539(3) Å
	b = 9.8305(4) Å
	c = 10.4233(5) Å
	α= 66.8870(10)°.
	β= 76.6150(10)°.
	γ = 83.6460(10)°.
Volume	710.72(5) $Å^3$
Z	2
Density (calculated)	1.818 Mg/m^3
Absorption coefficient	5.691 mm^{-1}
F(000)	380
Habitus, color	Colorless block
Crystal size	0.515 x 0.291 x 0.180 mm^3
Theta range for data collection	2.253 to 25.049°.
Index ranges	−9<=h<=9, −11<=k<=11, −12<=l<=12
Reflections collected	21423
Independent reflections	2513 [R(int) = 0.0325]
Completeness to theta = 25.049°	99.6 %
Absorption correction	Semi-empirical from equivalents
Max. and min. transmission	0.7452 and 0.4460
Refinement method	Full-matrix least-squares on F^2
Data / restraints / parameters	2513 / 6 / 181
Goodness-of-fit on F^2	1.092
Final R indices [I>2sigma(I)]	R1 = 0.0257, wR2 = 0.0734
R indices (all data)	R1 = 0.0267, wR2 = 0.0740
Largest diff. peak and hole	0.576 and -0.397 e.$Å^{-3}$

Table S3: Bond length and angles of 1

Bond Length [Å]		Angles [°]	
C(1)-N(1)	1.329(4)	N(1)-C(1)-C(2)	125.2(3)
C(1)-C(2)	1.385(4)	N(1)-C(1)-Br(1)	114.6(2)
C(1)-Br(1)	1.877(3)	C(2)-C(1)-Br(1)	120.1(2)
C(2)-C(3)	1.394(4)	C(1)-C(2)-C(3)	117.8(2)
C(3)-C(4)	1.399(4)	C(2)-C(3)-C(4)	118.1(2)
C(3)-C(6)	1.488(4)	C(2)-C(3)-C(6)	121.7(2)
C(4)-C(5)	1.383(4)	C(4)-C(3)-C(6)	120.0(2)
C(5)-N(1)	1.322(4)	C(5)-C(4)-C(3)	117.7(3)
C(5)-Br(2)	1.891(3)	N(1)-C(5)-C(4)	125.6(3)
C(6)-C(7)	1.402(4)	N(1)-C(5)-Br(2)	115.2(2)
C(6)-C(11)	1.403(4)	C(4)-C(5)-Br(2)	119.3(2)
C(7)-C(8)	1.388(4)	C(7)-C(6)-C(11)	119.9(2)
C(8)-C(9)	1.385(4)	C(7)-C(6)-C(3)	117.2(2)
C(9)-C(10)	1.384(4)	C(11)-C(6)-C(3)	122.9(2)
C(10)-C(11)	1.402(4)	C(8)-C(7)-C(6)	120.6(3)
C(11)-C(12)	1.489(4)	C(9)-C(8)-C(7)	119.9(3)
C(12)-C(13)	1.396(4)	C(10)-C(9)-C(8)	119.8(3)
C(12)-C(17)	1.399(4)	C(9)-C(10)-C(11)	121.6(3)
C(13)-C(14)	1.387(4)	C(10)-C(11)-C(6)	118.2(2)
C(14)-C(15)	1.386(5)	C(10)-C(11)-C(12)	117.9(2)
C(15)-C(16)	1.384(4)	C(6)-C(11)-C(12)	123.8(2)
C(16)-C(17)	1.387(4)	C(13)-C(12)-C(17)	118.9(3)
		C(13)-C(12)-C(11)	119.3(2)
		C(17)-C(12)-C(11)	121.7(2)
		C(14)-C(13)-C(12)	120.5(3)
		C(15)-C(14)-C(13)	120.2(3)
		C(16)-C(15)-C(14)	119.6(3)
		C(15)-C(16)-C(17)	120.7(3)
		C(16)-C(17)-C(12)	120.0(3)
		C(5)-N(1)-C(1)	115.5(2)

Symmetry transformations used to generate equivalent atoms: none.

32. NMR spectra

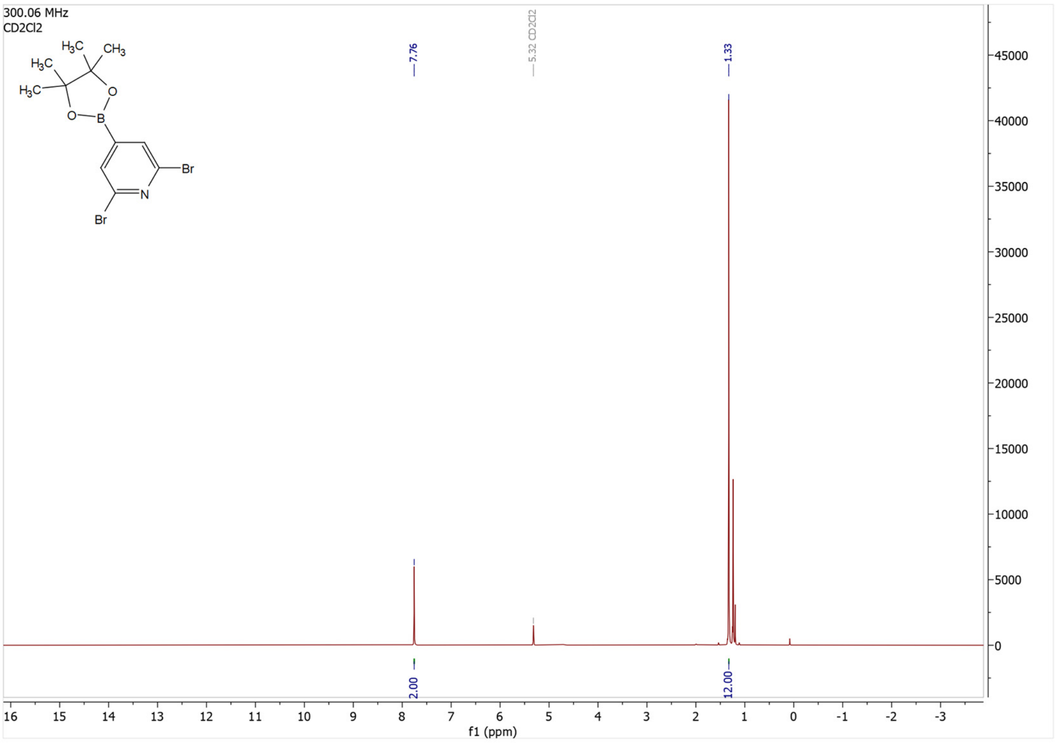

Figure S27. ^{1}H **NMR (300 MHz, 298 K,** CD_2Cl_2**) of M3.**

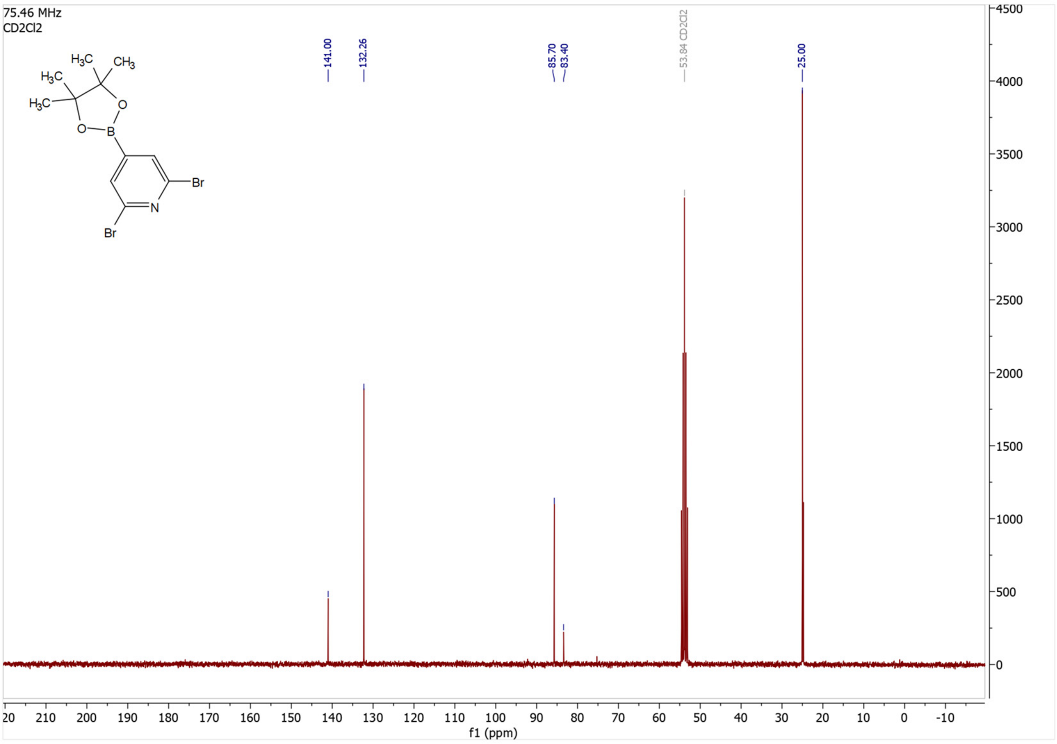

Figure S28. ^{13}C **NMR (75 MHz, 298 K,** CD_2Cl_2**) of M3.**

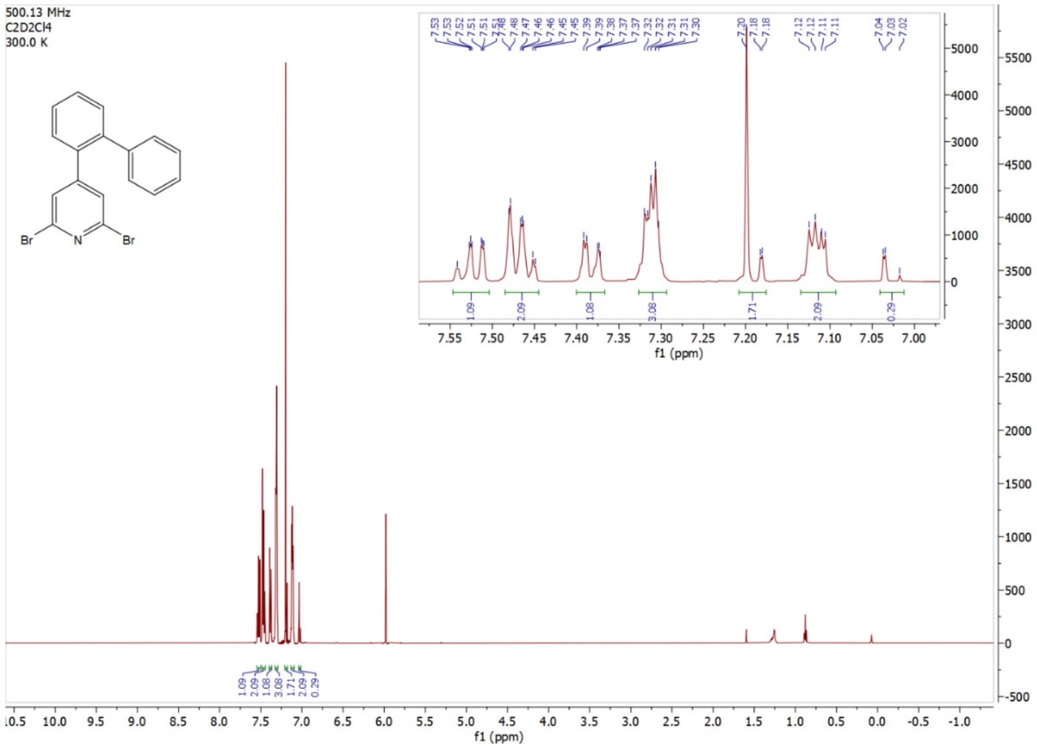

Figure S29. ^{1}H **NMR (500 MHz, 298 K,** $C_2D_2Cl_4$**) of 1.**

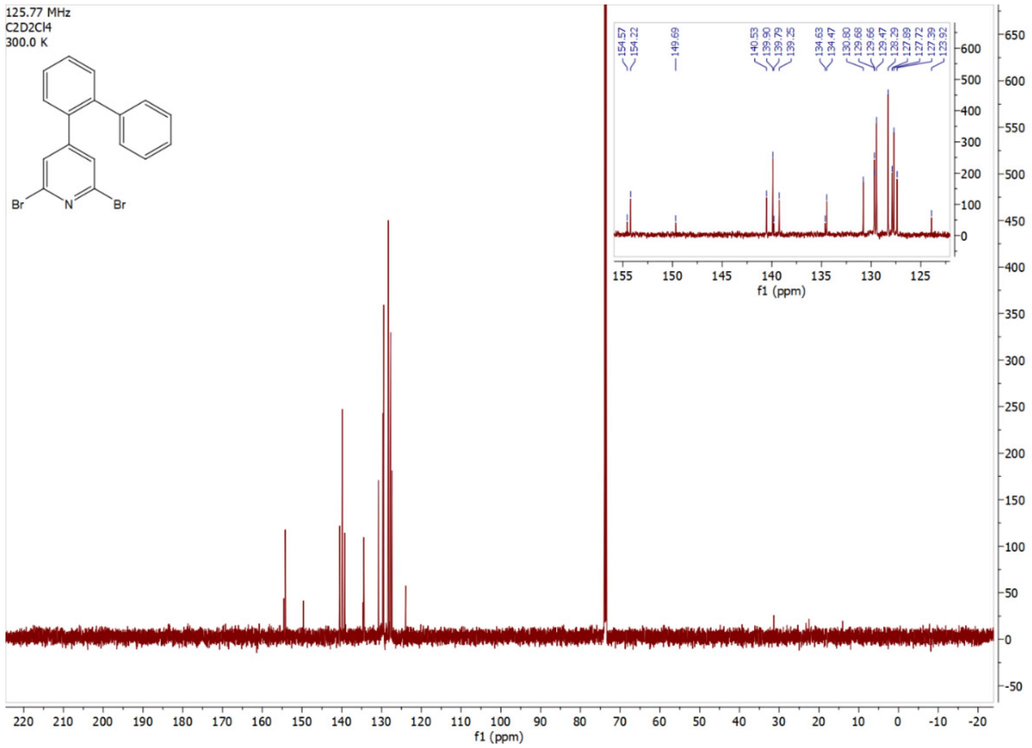

Figure S30. ^{13}C **NMR (125 MHz, 298 K,** $C_2D_2Cl_4$**) of 1.**

33. Mass spectra

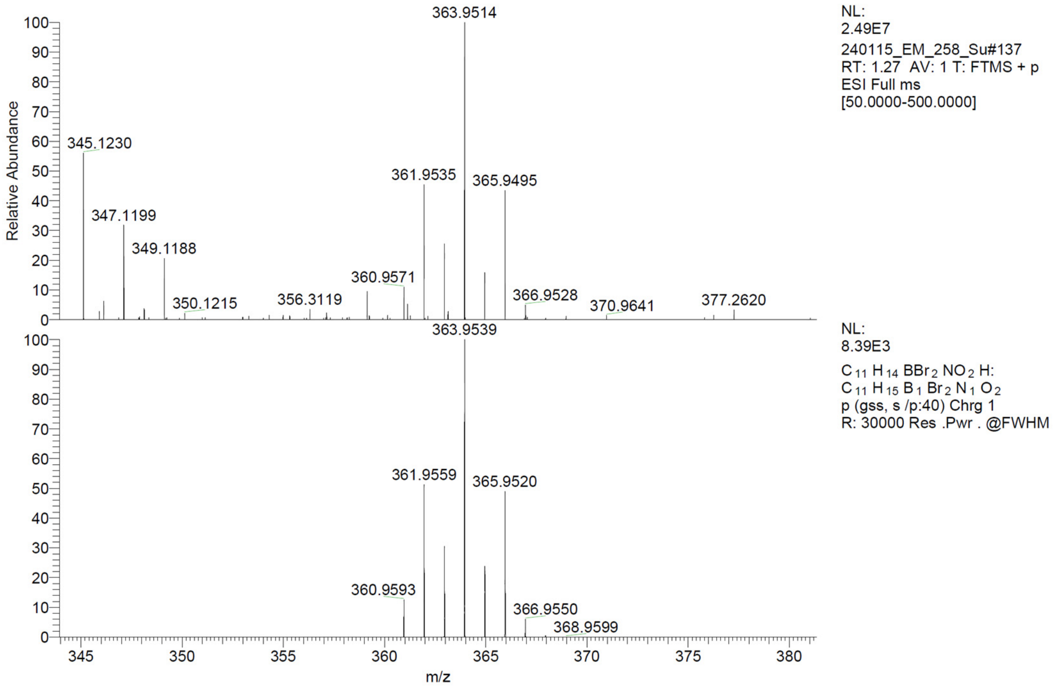

Figure S31. Mass spectrum (HR-ESI$^+$ taken from methanol) of M3.

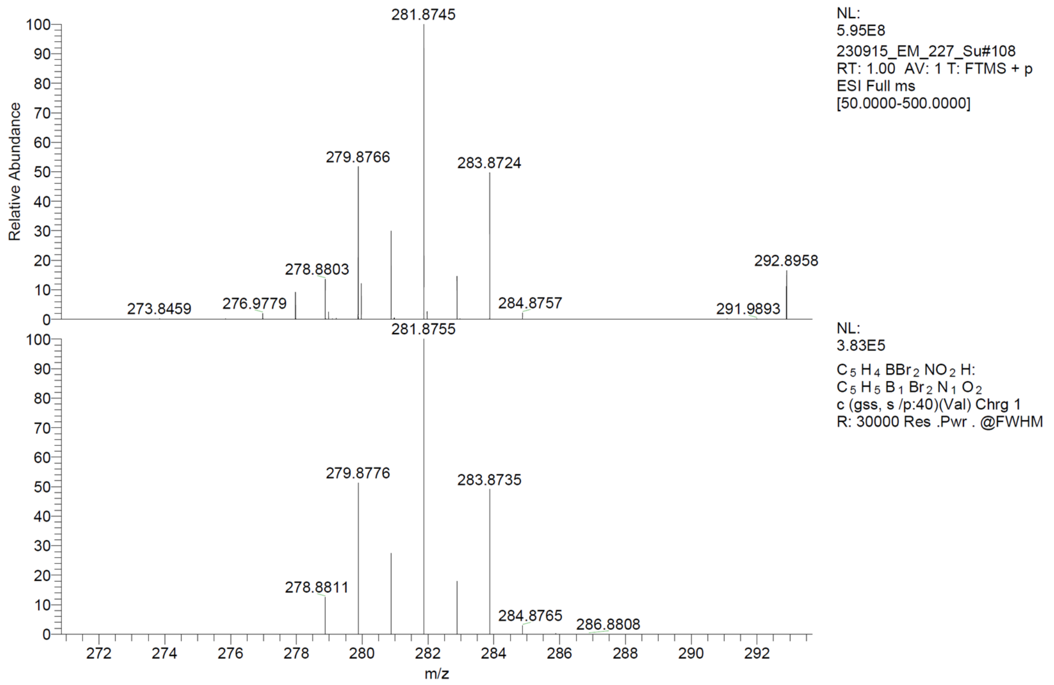

Figure S32. Mass spectrum (HR-ESI$^+$ taken from methanol) of M4.

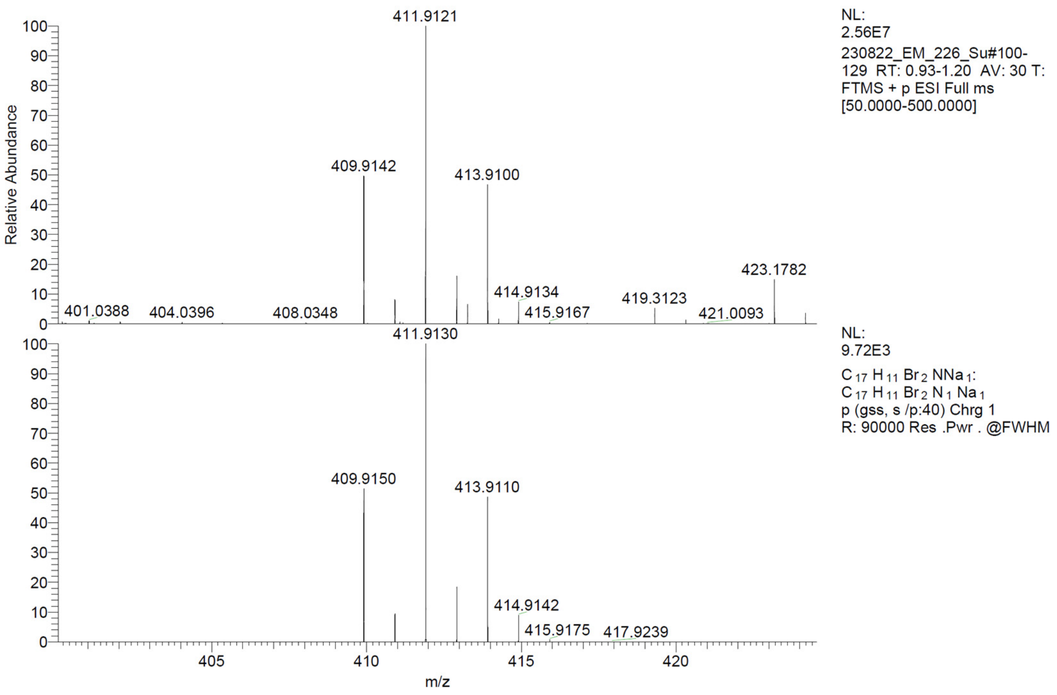

Figure S33. Mass spectrum (HR-ESI$^+$ taken from methanol) of 1.

References:

1. Kachel, S. R.; Klein, B. P.; Morbec, J. M.; Schöniger, M.; Hutter, M.; Schmid, M.; Kratzer, P.; Meyer, B.; Tonner, R.; Gottfried, J. M. Chemisorption and Physisorption at the Metal/Organic Interface: Bond Energies of Naphthalene and Azulene on Coinage Metal Surfaces. *J. Phys. Chem. C* **2020**, *124*, 8257-8268. DOI: 10.1021/acs.jpcc.0c00915.

2. Chen, L.; Rosen, J.; Björk, J. A Density Functional Benchmark for Dehydrogenation and Dehalogenation Reactions on Coinage Metal Surfaces. *ChemPhysChem* **2025**, *26*, e202400865. DOI: 10.1002/cphc.202400865.

3. Bannwarth, C.; Ehlert, S.; Grimme, S. GFN2-xTB—An Accurate and Broadly Parametrized Self-Consistent Tight-Binding Quantum Chemical Method with Multipole Electrostatics and Density-Dependent Dispersion Contributions. *J. Chem. Theory Comput.* **2019**, *15*, 1652-1671. DOI: 10.1021/acs.jctc.8b01176.

4. Bannwarth, C.; Caldeweyher, E.; Ehlert, S.; Hansen, A.; Pracht, P.; Seibert, J.; Spicher, S.; Grimme, S. Extended Tight-binding Quantum Chemistry Methods, WIREs Comput. Mol. Sci., 11, e1493. 2021.

5. Grimme, S. Exploration of Chemical Compound, Conformer, and Reaction Space with Meta-Dynamics Simulations Based on Tight-Binding Quantum Chemical Calculations. *J. Chem. Theory Comput.* **2019**, *15*, 2847-2862. DOI: 10.1021/acs.jctc.9b00143.

6. Becke, A. D. Density-functional exchange-energy approximation with correct asymptotic behavior. *Phys. Rev. A* **1988**, *38*, 3098-3100. DOI: 10.1103/PhysRevA.38.3098.

7. Becke, A. D. Density-functional thermochemistry. III. The role of exact exchange. *J. Chem. Phys* **1993**, *98*, 5648-5652. DOI: 10.1063/1.464913.

8. Lee, C.; Yang, W.; Parr, R. Accurate and simple analytic representation of the electron-gas correlation energy. *Phys. Rev. B* **1988**, *37*, 785-789.

9. Weigend, F.; Ahlrichs, R. Balanced basis sets of split valence, triple zeta valence and quadruple zeta valence quality for H to Rn: Design and assessment of accuracy. *Phys. Chem. Chem. Phys.* **2005**, *7*, 3297-3305, 10.1039/B508541A. DOI: 10.1039/B508541A.

10. Weigend, F. Accurate Coulomb-fitting basis sets for H to Rn. *Phys. Chem. Chem. Phys.* **2006**, *8*, 1057-1065, 10.1039/B515623H. DOI: 10.1039/B515623H.

11. Turbomole, V. 7.3, a development of University of Karlsruhe and Forschungszentrum Karlsruhe GmbH, 1989–2007. *TURBOMOLE GmbH, since* **2007**, *2010*.

12. Balasubramani, S. G.; Chen, G. P.; Coriani, S.; Diedenhofen, M.; Frank, M. S.; Franzke, Y. J.; Furche, F.; Grotjahn, R.; Harding, M. E.; Hättig, C.; et al. TURBOMOLE: Modular program suite for ab initio quantum-chemical and condensed-matter simulations. *J. Chem. Phys* **2020**, *152*. DOI: 10.1063/5.0004635.

13. Neese, F. Software update: the ORCA program system—version 5.0, WIREs Comput. Mol. Sci. 12 (2022) e1606.

14. Ishida, K.; Morokuma, K.; Komornicki, A. The intrinsic reaction coordinate. An ab initio calculation for HNC→HCN and H−+CH4→CH4+H− *J. Chem. Phys* **1977**, *66*, 2153-2156. DOI: 10.1063/1.434152.

15. Neese, F.; Wennmohs, F.; Hansen, A.; Becker, U. Efficient, approximate and parallel Hartree–Fock and hybrid DFT calculations. A 'chain-of-spheres' algorithm for the Hartree–

Fock exchange. *Chem. Phys.* **2009**, *356*, 98-109.

16. Izsák, R.; Neese, F. An overlap fitted chain of spheres exchange method. *J. Chem. Phys* **2011**, *135*. DOI: 10.1063/1.3646921.

17. Armarego, W. L. *Purification of laboratory chemicals*; Butterworth-Heinemann, 2017.

18. Sheldrick, G. Crystal structure refinement with SHELXL. *Acta Crystallographica Section C* **2015**, *71*, 3-8. DOI: doi:10.1107/S2053229614024218.

19. Hubschle, C. B.; Sheldrick, G. M.; Dittrich, B. ShelXle: a Qt graphical user interface for SHELXL. *J. Appl. Crystallogr.* **2011**, *44*, 1281-1284. DOI: doi:10.1107/S0021889811043202.

20. Spek, A. Platon/squeeze. *Acta Crystallogr., Sect. D: Biol. Crystallogr* **2009**, *65*, 148-155.

21. Putz, H.; Brandenburg, K. Diamond—Crystal and Molecular Structure Visualisation, 4.6. 4. *Crystal Impact: Bonn, Germany* **2020**.

22. Saito, N.; Takaya, J.; Iwasawa, N. Stabilized Gallylene in a Pincer-Type Ligand: Synthesis, Structure, and Reactivity of PGaIP-Ir Complexes. *Angew. Chem. Int. Ed.* **2019**, *58*, 9998-10002.
